# Clathrate Structure Determination by Combining Crystal Structure Prediction with Computational and Experimental ^129^Xe NMR Spectroscopy

**DOI:** 10.1002/chem.201604797

**Published:** 2017-02-14

**Authors:** Marcin Selent, Jonas Nyman, Juho Roukala, Marek Ilczyszyn, Raija Oilunkaniemi, Peter J. Bygrave, Risto Laitinen, Jukka Jokisaari, Graeme M. Day, Perttu Lantto

**Affiliations:** ^1^ NMR Research Unit Faculty of Science University of Oulu 90014 Oulu Finland; ^2^ Computational Systems Chemistry, School of Chemistry University of Southampton Southampton UK; ^3^ Faculty of Chemistry Wrocław University Joliot Curie 14 50-383 Wrocław Poland; ^4^ Laboratory of Inorganic Chemistry University of Oulu 90014 Oulu Finland

**Keywords:** computational chemistry, crystal structure prediction, density functional theory, first principles modelling, Xe NMR spectroscopy

## Abstract

An approach is presented for the structure determination of clathrates using NMR spectroscopy of enclathrated xenon to select from a set of predicted crystal structures. Crystal structure prediction methods have been used to generate an ensemble of putative structures of *o*‐ and *m*‐fluorophenol, whose previously unknown clathrate structures have been studied by ^129^Xe NMR spectroscopy. The high sensitivity of the ^129^Xe chemical shift tensor to the chemical environment and shape of the crystalline cavity makes it ideal as a probe for porous materials. The experimental powder NMR spectra can be used to directly confirm or reject hypothetical crystal structures generated by computational prediction, whose chemical shift tensors have been simulated using density functional theory. For each fluorophenol isomer one predicted crystal structure was found, whose measured and computed chemical shift tensors agree within experimental and computational error margins and these are thus proposed as the true fluorophenol xenon clathrate structures.

## Introduction

Over the past few years, the combined use of solid‐state nuclear magnetic resonance (NMR) and computational methods has developed into a practical method for determining crystal structures.^1^ This is due to the sensitivity of NMR chemical shifts to the molecular environment in a crystal, and the reliability of methods for predicting the relative NMR shielding of atoms in different environments. The approach involves first predicting the full set of low energy crystal structures available to a molecule, followed by chemical shielding calculations, which are matched against the measured chemical shifts of a material. Applications to organic crystals have found that, given a set of predicted crystal structures, the isotropic ^1^H chemical shifts are usually sufficient to identify which of the predicted structures corresponds to the material under investigation.^2^

Kazankin et al.^3^, ^4^ reported that xenon clathrates can be formed from several monosubstituted phenol compounds. With the exception of phenol,^5^ hydroquinone,^5^, ^6^, ^7^, ^8^ *p*‐cresol,^9^ and *p*‐fluorophenol,^10^ Xe clathrates have not been described in the English literature and have remained relatively unknown in the West. The crystal structures of *o*‐ and *m*‐fluorophenol xenon clathrates have, to our knowledge, never previously been determined. The NMR properties of Xe included in porous structures gives a potentially sensitive probe of the clathrate structure, which is explored here as a means of determining the structures of the *o*‐ and *m*‐fluorophenol xenon clathrates.

The first ^129^Xe NMR experiments were performed in 1951,^11^ but only a modest number of further studies appeared until the early 1980s when Ripmeester and Davidson investigated enclathrated xenon^12^ and Ito and Fraissard proposed the use of ^129^Xe NMR spectroscopy for probing the properties of zeolites.^13^ Today, the application range of ^129^Xe NMR spectroscopy extends from materials property studies of micro‐ and mesoporous solids through polymers, liquid crystals, proteins, and biosensors, to magnetic resonance imaging (MRI) for medical purposes.^14^, ^15^, ^16^, ^17^

The reason for using xenon to probe the properties of various materials arises from the high sensitivity of the shielding of ^129^Xe to its local environment, and that the variability in the shielding stems exclusively from environmental effects.^18^, ^19^, ^20^ Another reason is the good NMR receptivity of ^129^Xe, which is about 33 times that of ^13^C. A disadvantage in some cases may be the long ^129^Xe spin‐lattice relaxation time *T*_1_, which is, for example, several minutes in xenon clathrate hydrate.^12^ The application range of ^129^Xe NMR spectroscopy and MRI further widened when the so‐called optical pumping or hyperpolarization technique was invented,^21^ enabling the increase of the ^129^Xe polarization by up to four or five orders of magnitude and making experiments possible with very small amounts of xenon gas.

The work of Ripmeester and Davidson^12^ revealed the potential of ^129^Xe NMR spectroscopy in studies of clathrates. The ^129^Xe NMR spectrum of xenon clathrate hydrate consists of two broad resonance lines: one at 152±2 ppm and the other at 242±2 ppm downfield (smaller shielding) from the low pressure gas peak. The former resonance arises from xenon in large cages, and the latter from xenon in small cages. Thus, the ^129^Xe chemical shift relates to the size of cages.^22^ Furthermore, the resonance at 152 ppm displays a cylindrically symmetric powder lineshape with a chemical shift anisotropy of 32±3 ppm; the resonance lineshape relates to the shape of the cage accommodating the xenon atoms. The line intensities in turn relate to the occupation number. An illustrative example is the distribution of xenon atoms in the alpha cages of the NaA zeolite.^23^ The ^129^Xe NMR spectrum of xenon in NaA consists of several distinct signals, the chemical shift being determined by the occupation number of a cage.

Modeling of ^129^Xe NMR spectra of clathrate cages dates back to the studies of chemical shifts^24^ and NMR line shapes^25^ inside clathrate hydrate structures I and II, which were modeled as molecular clusters extracted from the clathrate structures. Similar quantum chemical cluster models were also applied in studies of Xe inside fullerene cages,^26^, ^27^ for which both relativistic^28^, ^29^ as well as dynamical and environmental^29^, ^30^ effects were studied. Current cluster modeling makes use of recent advances in the development of relativistic quantum chemistry methods, which have enabled very demanding studies of large heavy‐element systems such as cryptophanes^31^ and self‐organizing metallo‐supramolecular cages.^32^

Diffraction is the usual method for crystal structure solution. Structure determination of clathrates by diffraction methods can, however, be hindered by their instability. For the materials studied here, it was not possible to obtain a powder X‐ray diffraction (PXRD) pattern from the *m*‐fluorophenol clathrate and only a low resolution diffraction pattern could be obtained from the *o*‐fluorophenol clathrate, from which structure determination would not be possible. Therefore, ^129^Xe NMR spectra cannot be combined with PXRD experiments as usually done for identifying new clathrate phases.^33^, ^34^ The exceptional sensitivity of xenon's chemical shift anisotropy to its environment should enable the distinction between different candidate clathrate structures. This hypothesis was investigated by comparing experimentally observed and quantum chemically modeled ^129^Xe NMR isotropic and anisotropic shift parameters in clathrate structures obtained by computational crystal structure prediction (CSP).

CSP has until recently been focused primarily on predicting the single thermodynamically stable structure, or possibly a few low‐energy polymorphs. The lowest energy crystal structures available to a given molecule are, in all but very rare exceptions,^35^ close‐packed, leaving no room for the inclusion of guest molecules. There have been few reports of the prediction of porous molecular crystals^36^, ^37^, ^38^ or solvates.^39^, ^40^, ^41^ The empty host molecule frameworks of observed inclusion structures have been shown to often exist as local minima on the lattice energy surface, albeit sometimes at relatively high energies compared to close‐packed alternative structures.^41^, ^42^, ^43^ This suggests an efficient approach to the discovery of inclusion structures: searching for stable, empty frameworks using CSP methods and subsequently inserting the guest. This method should be particularly suited to weakly interacting guests such as Xe, where inclusion is expected to leave the host framework relatively unperturbed. The approach should be more efficient than searching the dramatically larger multi‐component phase space defined by the host and guest together.

In this study, CSP calculations were performed for *o*‐ and *m*‐fluorophenol with the specific aim of predicting realistic xenon clathrate complexes for which calculated ^129^Xe NMR parameters can be compared to measured spectra. Advanced first principles density functional theory (DFT) electronic structure calculations of the ^129^Xe NMR shielding tensors, with a proper inclusion of electron correlation as well as relativistic, periodic, and dynamical effects, have been carried out for a set of predicted structures. A comparison of the simulated isotropic and anisotropic Xe chemical shift parameters with experimentally observed solid state ^129^Xe NMR data allows the identification of the crystal structures of these elusive clathrates.

## Experimental Section

### NMR Spectroscopy

#### Preparation of samples

Clathrate samples were prepared from commercially available *o*‐ and *m*‐fluorophenol (Aldrich, 98 % assay) and used directly without purification. The substances were transferred into pyrex glass tubes (4 mm outer diameter, 0.8 mm walls) and connected to a volume‐calibrated vacuum line. Two cycles of freeze–thaw were applied to reduce oxygen content. Isotopically enriched ^129^Xe gas (Chemgas, 89 % enrichment) was then frozen into the evacuated tubes. The amount of transferred gas was controlled by the pressure drop in the vacuum line, measured with a digital pressure gauge (1 hPa precision). The amount of gas inserted into the tubes was chosen to be sufficient for saturation of a 3:1 host–guest clathrate stoichiometry and with excess to pressurize the sample tube so as to maintain clathrate structural stability at the desired experimental temperature of 250 K.^3^, ^4^ The glass sample tubes were flame sealed, cooled in liquid nitrogen, and equilibrated for a period of two weeks at ca. 243 K prior to NMR measurements.

#### NMR experiments

The ^129^Xe NMR spectrum of xenon in *m*‐fluorophenol was measured at 251 K on a Bruker DSX300WB spectrometer (^129^Xe Larmor frequency 83.03 MHz) using a 7 mm variable angle spinning (VAS) probe head (DOTY Scientific, Inc., USA), without spinning. The static powder spectrum (the axis of the solenoid coil was set perpendicular to the external magnetic field) was observed while applying cross‐polarization (CP) and proton decoupling. The following acquisition parameters were used: ^129^Xe pulse width 5 μs, ^1^H decoupling pulse width 16 μs, mixing time 5.4 ms, repetition time 35 s, strength of the ^1^H decoupling field 34 kHz, and number of collected free induction decay (FID) signals 220. The aim of applying proton decoupling was to diminish the effect of the ^129^Xe–^1^H dipolar coupling on the line width. The ^129^Xe chemical shift was measured relative to the isotropic ^129^Xe chemical shift in hydroquinone, in which the shift relative to zero‐pressure xenon is known to be 222.1 ppm. Temperature calibration was based on the measurement of the ^1^H chemical shift difference in a separate methanol sample.^44^ Prior to Fourier transformation the FID signal was multiplied by an exponentially decaying apodization function leading to ≈100 Hz line broadening.

The ^129^Xe NMR spectrum of xenon in *o*‐fluorophenol was in turn measured at 253 K on a Bruker DPX400 spectrometer (^129^Xe Larmor frequency 110.70 MHz) using a 5 mm broad band observe (BBO) probe head. ^129^Xe chemical shift is given with respect to the signal of zero‐pressure xenon gas, which was determined using two samples with known xenon pressure and extrapolation using the second virial coefficient. Temperature calibration was in this case performed using ^1^H chemical shifts in methanol, but with methanol placed in the annulus of a double tube system (outer tube 5 mm, inner tube 4 mm). A ^129^Xe pulse width of 29.5 μs was applied. Prior to Fourier transformation, the FID signal was multiplied by an exponentially decaying apodization function leading to 50 Hz line broadening.

The elements of the chemical shift tensors were determined in both cases using Dmfit.^45^ The uncertainties were estimated to be ±0.2 ppm, ±0.4 ppm, and <0.04, respectively, in the three adjusted NMR parameters: isotropic chemical shift (CS) δ≡δ_iso_=σrefiso −σ_iso_ referenced to the zero‐pressure limit Xe gas, chemical shift anisotropy (CSA) Δδ=δ_*zz*_−(δ_*xx*_+δ_*yy*_)/2 with positive/negative values for prolate/oblate spheroids along the *z*‐direction and asymmetry parameter η=(δ_*yy*_−δ_*xx*_)/(δ_*zz*_−δ_iso_) with the value 0 for an axially symmetric tensor (with respect to *z*) and 1 for the fully asymmetric case, when δ_*yy*_=δ_iso_. The isotropic Xe nuclear shielding constant is the trace of the shielding tensor, σ_iso_=(σ_*xx*_+σ_*yy*_+σ_*zz*_)/3. The components of the principal axis system (PAS) of the Xe shift tensor follow Haeberlen's convention:^46^ |δ_*zz*_−δ_iso_|≥|δ_*xx*_−δ_iso_|≥|δ_*yy*_−δ_iso_|. A relatively large contribution to the uncertainties arises from the wavy background in the experimental spectra, which was therefore eliminated.

### Powder X‐ray diffraction

Samples of both *o*‐ and *m*‐fluorophenol were prepared in glass capillaries. Xenon gas was frozen into the evacuated capillaries, which were flame‐sealed after submerging in liquid N_2_. Capillaries containing the samples were kept immersed in liquid N_2_ prior to mounting in a pre‐cooled single‐crystal diffractometer. Diffraction data were collected at 100 K using graphite monochromated Mo_Kα_ radiation.

### Crystal structure prediction

Crystal structure prediction (CSP) calculations involved five steps: i) the lowest energy conformations of the isolated molecules were identified; ii) hypothetical crystal packings were generated and then lattice energy minimized using a simple force field and rigid‐molecule constraints; iii) a subset of the lowest energy predicted crystal structures were re‐optimized using an anisotropic atom–atom force field; iv) intramolecular flexibility was introduced, allowing the hydroxyl group to reorient in response to packing forces in each low energy crystal structure; v) porous structures were identified, xenon atoms inserted into the pores, and the structures once again re‐optimized.

**Figure 1:**
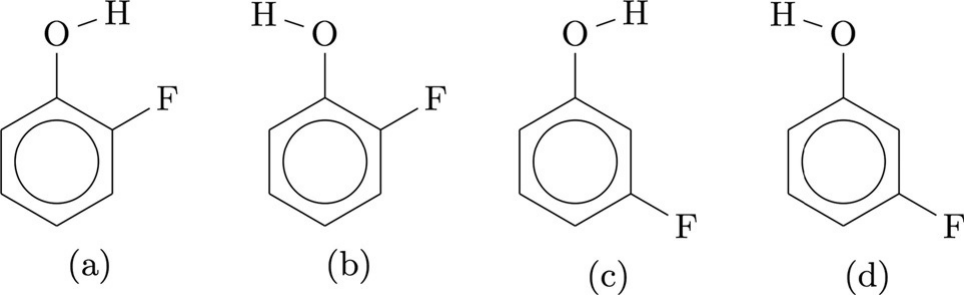
Conformers of the two fluorophenol isomers. a) *cis*‐ and b) *trans‐o*‐fluorophenol c) *cis*‐ and d) *trans‐m*‐fluorophenol.

Molecular geometries, energies, and charge densities, calculated by DFT using Gaussian 09^47^ with the B3LYP^48^, ^49^ functional and 6‐311G(d,p) basis set, were used throughout the CSP calculations. Two stable, planar conformers of each molecule were identified (Figure 1) and DFT predicts that their energies are sufficiently close that either could form low energy crystal structures.^50^ Therefore, both conformers of both molecules were included in the CSP study.

Hypothetical crystal structures were generated with the rigid DFT‐optimized molecular geometries using Monte Carlo simulated annealing^51^ with Materials Studio. Searches were performed in the most commonly observed space groups of known molecular organic crystal structures; 25 space groups were searched with one molecule in the asymmetric unit (*Z*′=1) and 5 space groups with *Z*′=2, including all combinations of the two conformers in the asymmetric unit.

Structures with a lattice energy within a 15 kJ mol^−1^ window from the lowest energy structure were further refined with an anisotropic, atomic multipole‐based intermolecular atom–atom potential model,^52^ combined with a DFT treatment of intramolecular energies and geometries. Duplicate crystal structures were removed using the Compack^53^ program and the resulting unique structures were re‐optimized using the CrystalOptimizer^54^ program to treat flexibility of the hydroxyl group within each crystal structure. Full details of the conformational analysis and CSP methods are provided in the Supporting Information.

### Selection of likely clathrate host structures

Lattice energy differences between polymorphs are usually very small and rarely exceed 8 kJ mol^−1^.^55^ Since voids in crystal structures are thermodynamically unfavorable,^42^, ^56^ structures within a larger energy range than in the usual application of CSP to polymorph prediction were considered as putative inclusion frameworks. Crystal structures within an energy cutoff of 13 kJ mol^−1^ above the global minimum for each molecule were considered.

To guide the selection of potential clathrate host frameworks, the guest‐to‐host volume ratio *R_g_* in 31 representative clathrate structures, taken from the Cambridge Structural Database (CSD), were examined (see Supporting Information for details). The volume ratio *R_g_* is calculated with Equation (1):

(1) $$R_{g}=100\frac{V_{g}}{V_{H}}\left[\%\right]$$

where *V_g_* is the van der Waals volume of the guest molecule and *V_H_* is an individual void's contact volume.^57^

One xenon atom was inserted at each cavity's centroid coordinates and the resulting xenon clathrate structures were geometry‐optimized as described above, assuming rigid molecules, imposing no space group symmetry and using an ad hoc exp‐6 potential for xenon.

### ^129^Xe NMR shielding calculations

The three ^129^Xe NMR parameters of the predicted structures were modeled and compared with the experimental data in several stages. In NMR modeling the chemical shift reference is a free Xe atom. In the first step, all predicted clathrate structures were subjected to nonrelativistic (NR) NMR modeling of cluster models. The results from the NR cluster models were used to identify likely candidates for the experimentally observed clathrate structures. These candidates were then further studied using DFT calculations on their fully periodic models. Finally, a few crystal structures that agreed best with experimental NMR results were chosen for more detailed modeling. Full details are given in the Supporting Information.

#### Screening of structures by cluster modeling

The NMR parameters of probable clathrate structures were first modeled using clusters consisting of a single xenon‐occupied cavity, including the Xe atom and all nearby fluorophenol molecules. Nonrelativistic Xe shielding tensor calculations were performed on the cluster models using Turbomole.^58^ The BHandHLYP^59^, ^60^ hybrid functional, including 50 % of exact Hartree–Fock (HF) exchange (EEX), was chosen based on benchmark calculations on the xenon–benzene system, where high quality nonrelativistic ab initio Xe chemical shifts are reproduced reasonably well.^29^ BHandHLYP has been shown to slightly underestimate Xe chemical shift and anisotropy, leaving room for improvements in the modeling by approaching the experimental values from below for Xe‐containing molecules,^61^, ^62^, ^63^ Xe atoms moving freely inside buckminster fullerenes,^29^, ^30^ and in self‐organizing metallo‐supramolecular cages.^32^ Those studies show that the typical overestimation of Xe CS and CSA by pure generalized gradient approximation (GGA) DFT functionals, such as PBE^64^ and BLYP,^59^, ^65^ is partly compensated when the amount of exact HF exchange is increased in B3LYP (EEX=20 %) and BHandHLYP (EEX=50 %) hybrid functionals. Calculations have been performed with a mixed basis set denoted as MHA/SVP (see Supporting Information for details).

#### Periodic modeling of likely candidates

Five *o*‐fluorophenol and seven *m*‐fluorophenols crystal structures, with screening level NMR parameters close to the experimental values, were chosen for further scrutiny. These structures were optimized with respect to both atomic positions and lattice parameters with planewave, periodic DFT with the PBE functional and Tkatchenko–Scheffler (TS) dispersion correction,^66^ denoted as PBE‐TS structures from now on.

Following optimization, NMR shielding tensors were computed with the PBE functional using the gauge‐including projector augmented wave (GIPAW) method^67^, ^68^ (see Figures 6 and 7 below). These periodic DFT results include the scalar relativistic (SR) effects on Xe shielding at the 1‐component zeroth‐order regular approximation (ZORA)^69^, ^70^ level of theory. Details of the periodic calculations with CASTEP^71^, ^72^ are in the Supporting Information.

#### Detailed modeling of the most probable structures

After periodic NMR calculations of the DFT optimized structures, the most probable clathrate structures were selected for more detailed DFT NMR modeling using the Amsterdam Density Functional (ADF) program.^73^, ^74^ In addition to the NR and SR‐ZORA quantum chemistry, ADF provides a 2‐component spin‐orbit SO‐ZORA method also including SR effects.^69^, ^75^

A much wider range of DFT functionals than is currently available in periodic CASTEP can be used in ADF. Calculations were performed with the jcpl/TZP mixed basis set^76^ (see Supporting Information for details) on clusters comprising a single cavity to test the influence of correlation treatment and the amount of EEX on NMR parameters using the PBE, BLYP, B3LYP and BHandHLYP functionals at the SR‐ZORA level. By using the results in scaling of the periodic PBE results, estimates of SR periodic BLYP, B3LYP, and BHandHLYP results were obtained. It is expected that, as the electron correlation description is improved by an increasing portion of EEX, the calculated NMR parameters for the true crystal structures should approach the experimental results. Hence, the BHandHLYP‐scaled periodic PBE results provide the best estimation of ^129^Xe NMR parameters and lack only contributions due to molecular dynamics and relativistic SO effects. The latter were treated as an additive correction obtained from the difference of the static SR‐ and SO‐ZORA cluster calculations with the BHandHLYP functional.

The effect of Xe dynamics at *T=*300 K for ^129^Xe NMR shielding parameters was modeled for the few most probable clathrates by canonical NVT Metropolis Monte Carlo (MC‐NVT) of Xe motion on a potential energy surface inside a cluster cavity with fixed PBE‐TS optimized geometry. The temperature effects on Xe chemical shift and anisotropy were calculated as the difference between the thermally averaged shielding tensor and the reference tensor with Xe at the center of the cage.

For further details, see the Supporting Information, which includes a detailed description of crystal structure prediction (CSP) and its performance for the known high density crystal forms of the two fluorophenols, as well as the method to compute guest to host volume ratios. Revised Williams99 parameters for H…A interactions, as well as potential parameters for F and Xe are described. Also included are: details of DFT structure optimization, Xe NMR calculations, and modeling of Xe dynamics; Xe NMR results at different levels and their sensitivity to structure; details of powder X‐ray diffraction (PXRD) measurements and results, and a number of clathrate structures in CIF format.

## Results and Discussion

### Experimental NMR results

Both isomers formed clathrates with xenon under the experimental conditions and allowed recording of properly shaped powder patterns without active mixing and crushing of crystals during their formation. A powder‐like appearance and lack of macroscopic crystallites was confirmed by visual inspection and the fit to spectra. There is no preferential growth imposed by tube walls as this would not allow for proper random averaging of orientations of crystallites in relation to the magnetic field of the spectrometer.

**Figure 2:**
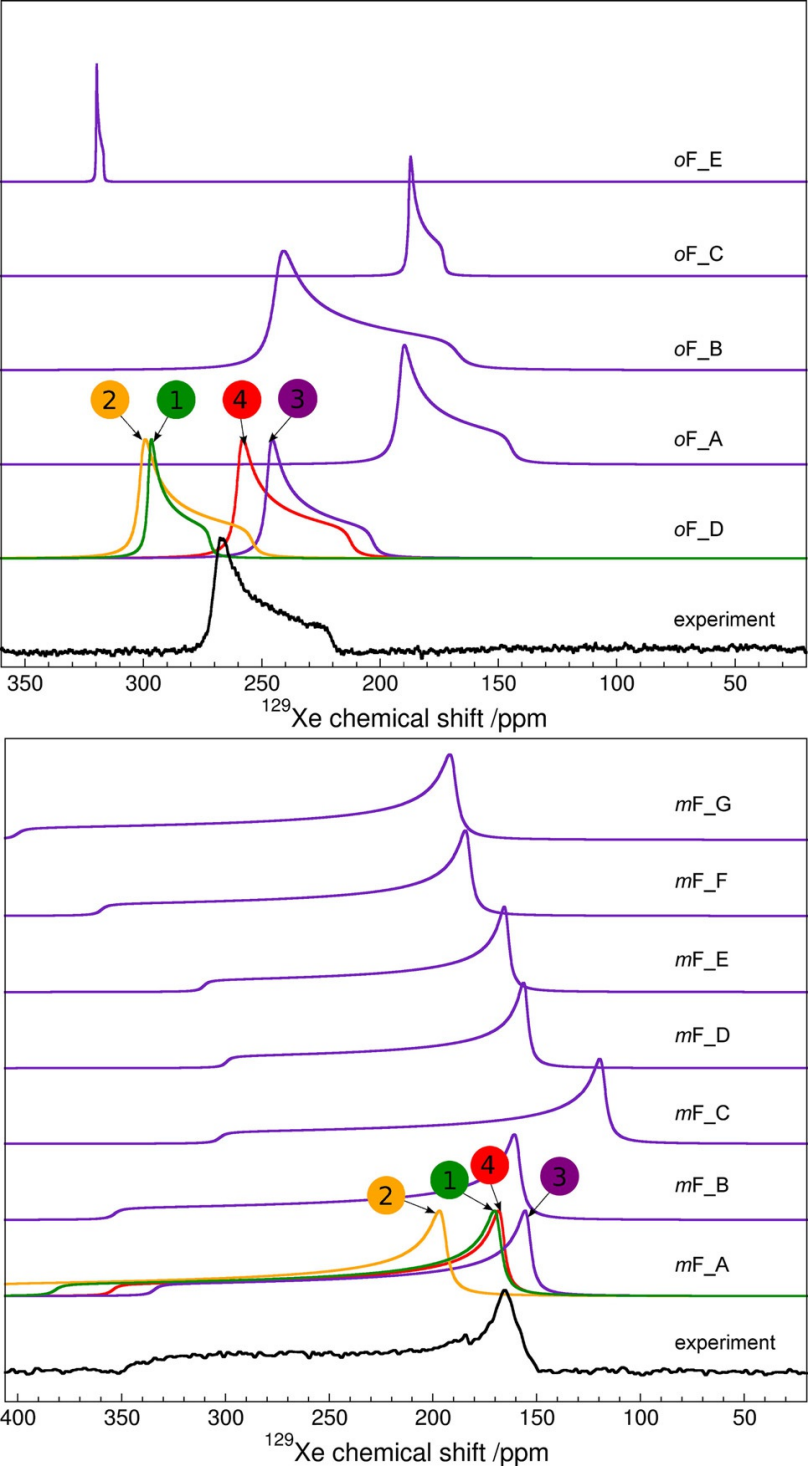
Experimental (black) and calculated (indigo) ^129^Xe NMR powder spectra of *o*‐fluorophenol (top) and *m*‐fluorophenol (bottom) with ^129^Xe gas reference at zero. Calculated spectra for all structures were obtained from BHandHLYP‐scaled periodic PBE NMR parameters at PBE‐TS optimized geometry (method/structure=BHandHLYP/PBE‐TS). For the most probable candidates, *o*F_D and *m*F_A, results from different periodic modeling levels are displayed: (1, green) PBE/CSP, (2, orange) PBE/PBE‐TS, (3, indigo) BHandHLYP/PBE‐TS and (4, red) BHandHLYP/PBE‐TS with effects due to Xe dynamics at *T=*300 K (see text for details).

Axially symmetric powder spectra have been observed for both clathrates (Figure 2), which confirms the existence of highly symmetric voids occupied by xenon atoms. High values of ^129^Xe chemical shift for both clathrates, δ=228.5 ppm for *m*‐fluorophenol and δ=256.0 ppm for *o*‐fluorophenol, suggest tight cages with only one occupational site. In such cases, where ^129^Xe NMR powder patterns are expected to reflect only interactions of xenon atoms with the immediately adjacent host molecules, the influence of neighboring cages with xenon is expected to be negligible.^77^

The spectrum of *m*‐fluorophenol (Figure 2) resembles that of β‐hydroquinone,^8^ β‐phenol, and *p*‐fluorophenol, with one notable difference; it has the largest observed value of ^129^Xe NMR CSA among known inclusion compounds: Δδ=183.3 ppm versus 161.9, 171, and 164 ppm for hydroquinone,^78^ phenol,^5^ and *p*‐fluorophenol,^79^ respectively. Small additional features in the observed spectrum of *m*‐fluorophenol are attributed to amorphous host substance and xenon atoms adsorbed in this amorphous phase.

The ^129^Xe NMR spectrum of *o*‐fluorophenol shows negative CSA (Figure 2), Δδ=−47.5 ppm, in contrast to all previously known solid‐state ^129^Xe NMR powder patterns of phenol clathrates for which positive CSA is observed.^5^, ^6^, ^7^, ^8^, ^9^, ^10^ Changes in the sign of the CSA can be observed in a group of porous channel‐like dipeptides,^80^ where CSA changes from positive to negative with increasing xenon gas pressure, and therefore with pronounced Xe–Xe interactions playing a greater role. No changes of the NMR spectrum of *o*‐fluorophenol clathrate have been observed when repeating the experiment with samples at different pressures. The small negative CSA may result from an axially symmetric but oblate environment around the xenon atom in this clathrate.

### Powder X‐ray diffraction results

A PXRD pattern could not be obtained from *m*‐fluorophenol clathrate, which transformed too quickly to the known high density form when transferred to the diffractometer. This is confirmed by comparison of the measured PXRD pattern to that simulated from the known crystal structure. For *o*‐fluorophenol, a broad powder pattern was obtained that does not correspond to either known crystal structures (see Supporting Information). The pattern is too broad to index, so it could not be used to determine the structure.

### Crystal structure prediction results

**Figure 3:**
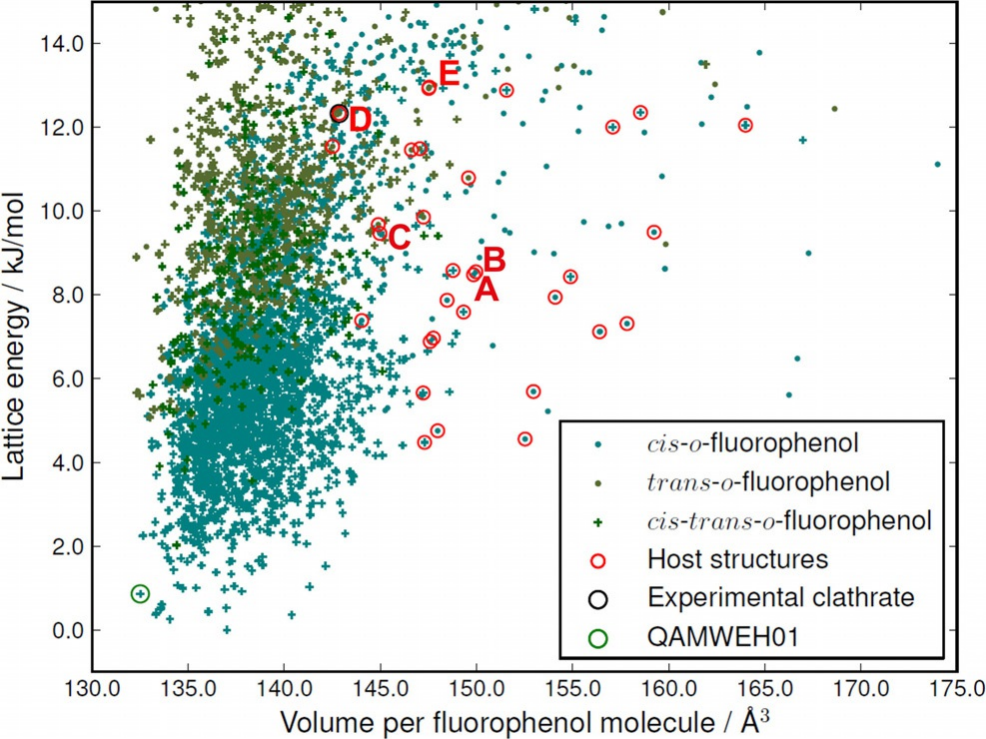
The crystal energy landscape of *o*‐fluorophenol. Each point represents one crystal structure and is colored according to the molecular conformation (*cis‐trans‐o*‐fluorophenol refers to *Z*′=2 structures containing both conformers). Structures with a lattice energy within 13 kJ mol^−1^ from the lowest energy structure and having cavities of suitable size for xenon absorption (see text) are encircled in red. The experimentally known high‐pressure polymorph (CSD refcode QAMWEH01) is encircled in green. The labels A–E correspond to structures *o*F_A to *o*F_E in the text.

**Figure 4:**
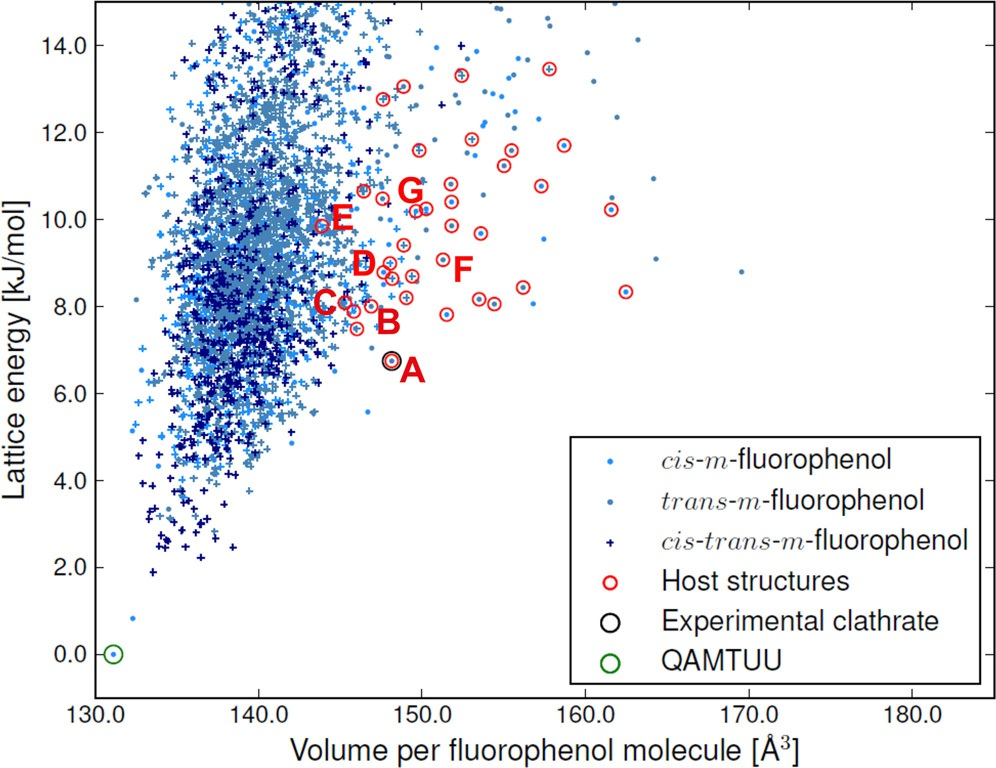
The crystal energy landscape of *m*‐fluorophenol. Each point represents one crystal structure and is colored according to the molecular conformation (*cis‐trans‐m*‐fluorophenol refers to *Z*′=2 structures containing both conformers). Structures with a lattice energy within 13 kJ mol^−1^ from the lowest energy structure and having cavities of suitable size for xenon absorption (see text) are encircled in red. The experimentally known stable polymorph (CSD refcode QAMTUU) is encircled in green. The labels A–G correspond to structures *m*F_A to *m*F_G in the text.

CSP resulted in an exceptionally large number of low energy crystal structures for each molecule (Figures 3 and 4).

For *o*‐fluorophenol, *Z*′=2 crystal structures with both molecules in the *cis*‐conformation are energetically favored. The known^81^ high‐pressure polymorph II (CSD refcode QAMWEH01) is located in the search, 0.8 kJ mol^−1^ above the global minimum (Figure 3) and with a slightly higher density, as expected for a high pressure polymorph. The known low‐temperature polymorph I^81^ (CSD refcode QAMWEH) is a disordered Z′=1.5 structure and could not be predicted with the methods employed here.

One crystal structure of *m*‐fluorophenol is known^81^ (CSD refcode QAMTUU) and corresponds to the global lattice energy minimum from the search. The structure is geometrically well reproduced (with a deviation in atomic positions in a 15‐molecule cluster taken from predicted and X‐ray crystal structures of RMSD_15_=0.203 Å).

Overlays of both matches are included in the Supporting Information. The successful reproduction of the known crystal structures of both molecules amongst the lowest energy predicted structures provides confidence in the sampling of crystal structures and of their lattice energy rankings.

### Selection rules for clathrate structures

Because the inclusion of guest molecules in clathrates can significantly stabilize the structure, using the lattice energy of the empty host alone is not useful for selection of promising structures. The analysis of known clathrates found that the guest‐to‐host volume ratio *R_g_* is normally distributed with a mean of 59 % and a standard deviation of 8 percentage units, in good agreement with Rebek's “55 % solution”^82^ and providing further evidence for the empirical rule that *R_g_* in observed inclusion structures should fall in this limited range.^82^, ^83^

Of the predicted crystal structures within 13 kJ mol^−1^ of the global minimum for *o*‐fluorophenol and *m*‐fluorophenol, 230 and 223 are porous (having cavities >10 Å^3^). 33 (*o*‐fluorophenol) and 32 (*m*‐fluorophenol) of the predicted structures have cavities of a suitable volume to accommodate xenon as a guest, which were take to be those with *R_g_* within three standard deviations (±3σ) for observed clathrates. These structures are encircled in red in Figures 3 and 4. The lowest energy of these structures are 4.5 and 6.7 kJ mol^−1^ above the global minima for *o*‐ and *m*‐fluorophenol, respectively.

### Computational NMR results

#### Screening of structures by cluster models

**Figure 5:**
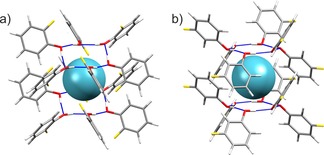
Examples of clusters used in the initial screening NMR calculations of the predicted clathrate structures. The clusters shown correspond to the structures selected as best models of the observed clathrates: a) *o*‐fluorophenol structure *o*F_D, b) *m*‐fluorophenol structure *m*F_A. Atoms are colored by element: carbon (grey); oxygen (red); hydrogen (white); fluorine (yellow).

**Figure 6:**
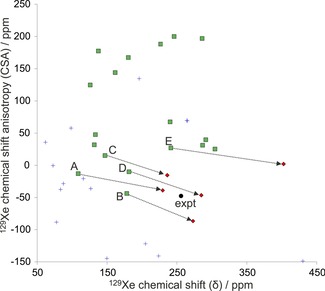
^129^Xe NMR parameters computed at screening (nonrelativistic DFT/BHandHLYP/MHA) level for cluster models of the predicted clathrate structures of *o*‐fluorophenol. Structures with asymmetry η>0.04 are shown as blue crosses, whereas structures with symmetric chemical shift tensors (η≤0.04) are shown as green squares. The experimental data (expt, black filled circle) is included for comparison. Structures that were considered for further study are connected with the black arrows to the periodic GIPAW/PBE calculation of Xe shielding from periodic PBE‐TS optimized structures of the corresponding crystal (red diamonds). The labels A–E correspond to structures *o*F_A to *o*F_E in the text.

**Figure 7:**
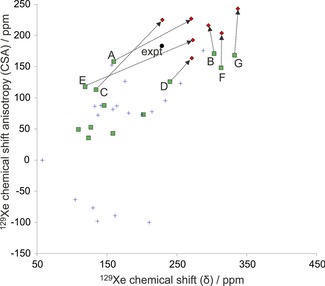
^129^Xe NMR parameters computed at screening (nonrelativistic DFT/BHandHLYP/MHA) level for cluster models of the predicted clathrate structures of *m*‐fluorophenol. Structures with asymmetry η>0.04 are shown as blue crosses, whereas structures with symmetric chemical shift tensors (η≤0.04) are shown as green squares. The experimental data (expt, black filled circle) is included for comparison. Structures that were considered for further study are connected with the black arrows to the periodic GIPAW/PBE calculation of Xe shielding from periodic PBE‐TS optimized structures of the corresponding crystal (diamonds). The labels A–G correspond to structures *m*F_A to *m*F_G in the text.

Examples of cluster models used for initial screening are shown in Figure 5. As can be seen in Figures 6 and 7, even for a set of structures with similar void volumes and packing energies, the computed NMR tensor parameters for ^129^Xe vary widely in the initial screening using cluster models of the predicted structures. This demonstrates the exceptional sensitivity of the ^129^Xe NMR parameters to small differences in cavity size and shape.

Calculated NMR parameters for the 33 *o*‐ and 32 *m*‐fluorophenol clathrate structures obtained with nonrelativistic DFT cluster models are tabulated in Tables S4 and S5 (Supporting Information). Many of these structures, including all predicted *Z*′=2 structures, have chemical shift tensors with significant asymmetry; these voids are clearly not compatible with the experimental spectra, in which η<0.04. At this stage, only the structures with axially symmetric chemical shift tensors (green squares in Figures 6 and 7) are kept as possible candidates for the experimental structures.

Optimization of the clathrate structure after insertion of the xenon into the host allowed the structures to relax in response to guest insertion. Despite very small structural changes, this significantly affected the calculated NMR parameters (Figure S7 in the Supporting Information).

Because the screening level of theory is expected to underestimate both CS and CSA by a few tens of ppm, structures with both properties of the correct sign and underestimated were focused on, also including a few structures with overestimated CS for *m*‐fluorophenol and slightly positive CSA for *o*‐fluorophenol, for higher level calculations. This excluded all but five *o*‐fluorophenol and seven *m*‐fluorophenol structures; hereafter, these structures are referred to as *o*F_A to *o*F_E (in order of increasing energy, Figure 3) and *m*F_A to *m*F_G (Figure 4). All of these candidate clathrate structures have space group symmetry *R* $\bar{3}$ .

#### NMR chemical shift parameters from periodic DFT calculations

NMR shielding tensors were calculated by periodic DFT for the five plausible *o*‐fluorophenol and seven *m*‐fluorophenol clathrate structures. Periodic modeling was found to be necessary and the geometry relaxation with periodic PBE‐TS shifts the CS and CSA towards larger magnitudes, corresponding to more elliptic, prolate (Δδ>0) and oblate (Δδ<0) *m*‐ and *o*‐fluorophenol cavities, respectively.

NMR parameters from SR GIPAW/PBE calculations at the PBE‐TS optimized geometries are tabulated in Tables S6 and S7 in the Supporting Information, and are displayed in Figures 6 and 7 as red diamonds connected with arrows to the corresponding screening cluster result (green squares). It should be noted that the periodic results set upper limits on ^129^Xe CS and CSA, as the PBE functional (and all pure GGA DFT functionals) overestimates the magnitudes of both parameters.^29^, ^32^, ^61^, ^62^, ^63^ Although the disagreement in CS and CSA of some of the candidate structures now makes them unlikely (such as *o*F_C, *o*F_E, and *m*F_G), all were kept for further analysis with more detailed NMR calculations.

#### Results of detailed NMR calculation of the most likely candidates

Typically, the crystal lattice effect, that is, the difference of ^129^Xe NMR parameters between the periodic and cluster models of the same PBE‐TS optimized clathrate, increases both CS and CSA as seen in Tables 1 and 2. The extent of the change is, however, very case‐specific and unforeseeable, which makes periodic modeling essential, in one form or another, in the present search for a best candidate of an unknown clathrate stucture.

**Table 1:** Theoretically modelled ^129^Xe NMR chemical shift (δ) and anisotropy (CSA, Δδ) in ppm for the five most likely *o*‐fluorophenol CSP candidates in their PBE‐TS optimized structures, unless stated otherwise.

	*o*F_A		*o*F_B		*o*F_C		*o*F_D		*o*F_E	
Method	δ	Δδ	δ	Δδ	δ	Δδ	δ	Δδ	δ	Δδ
*Cluster model* ^[a]^										
PBE	218.7	−14.3	245.0	−79.3	215.1	4.6	261.5	−38.6	370.5	7.7
BLYP	226.5	−14.6	253.4	−81.8	222.5	4.1	270.9	−40.2	381.5	7.9
B3LYP	195.9	23.1	223.1	−75.6	195.9	0.9	240.8	−36.6	338.1	13.1
BHandHLYP	165.8	−21.3	190.7	−68.3	162.6	−1.2	209.9	−35.8	288.5	18.3
BHandHLYP^[b]^	145.8	−20.5	175.0	−61.8	143.2	0.9	189.4	−31.6	262.0	14.4
BHandHLYP^[c]^	174.9	−11.6	192.3	−71.2	171.3	6.7	216.7	−31.6	292.0	31.1
*Periodic model* ^[d]^										
PBE^[e]^	195.1	−28.1	272.1	−66.4	238.7	7.0	288.8	−26.2	362.1	23.1
PBE	229.8	−38.9	273.2	−86.7	236.4	−15.4	285.0	−46.5	402.7	2.0
BLYP^[f]^	237.3	−39.1	281.5	−89.3	243.6	−15.9	294.3	−48.1	413.6	2.1
B3LYP^[f]^	206.3	−48.0	250.8	−82.9	216.7	−19.3	263.8	−44.5	369.5	7.5
BHandHLYP^[f]^	175.6	−46.3	217.7	−75.4	182.6	−21.5	232.3	−43.6	318.8	12.7
BHandHLYP^[g]^			236.2	−82.4			243.7	−46.8		
BHandHLYP^[h]^			237.8	−85.4			250.5	−42.6		
Experimental							256.0	−47.5		

[a] Scalar relativistic SR‐ZORA calculations of a cluster model of one clathrate cavity with ADF code^73^, ^74^ using Xe/other=jcpl/TZP basis sets.^76^ ^129^Xe chemical shift (δ=σ_Xe−atom_−σ) with respect to free Xe atom shielding values (σ_Xe−atom_): PBE/BLYP/B3LYP/BHandHLYP=5752.2/5752.6/5752.1/5752.0 ppm. Δδ (CSA) defined in principal axis system (PAS) along the unique axis (perpendicular to the plane of O−H rings). Asymmetry parameter η=0 in all cavities due to the cylindrical symmetry. [b] As footnote [a], but at nonrelativistic (NR) level of theory with σ_Xe−atom_(BHandHLYP)=5643.4 ppm. [c] As footnote [a], but at relativistic spin‐orbit SO‐ZORA level of theory with σ_Xe−atom_(BHandHLYP)=6609.3 ppm. [d] Scalar relativistic GIPAW results obtained with CASTEP code.^71^, ^72^ All periodic ^129^Xe chemical shifts referenced to CASTEP/PBE value: σ_Xe−atom_=5926.3 ppm. [e] In the CSP optimized crystal geometry. [f] As footnote [d], but estimated by scaling GIPAW/PBE PAS components with factors obtained at SR‐ZORA level for cluster model resulting in data in footnote [a]. [g] Correction added to result of footnote [f] due to thermal averaging (AVE) over Xe motion at *T=*300 by MC‐NVT simulation (see text for details). [h] Relativistic spin‐orbit (SO) corrections obtained as difference of SO‐ZORA and SR‐ZORA calculations with ADF code added to result of footnote [g].

**Table 2:** Theoretically modelled ^129^Xe NMR chemical shift (δ) and anisotropy (CSA, Δδ) in ppm for the seven most likely *m*‐fluorophenol CSP candidates in their PBE‐TS optimized structures, unless stated otherwise.

	*m*F_A		*m*F_B		*m*F_C		*m*F_D		*m*F_E		*m*F_F		*m*F_G	
Method	δ	Δδ	δ	Δδ	δ	Δδ	δ	Δδ	δ	Δδ	δ	Δδ	δ	Δδ
*Cluster model* ^[a]^														
PBE	259.6	219.9	272.1	188.5	216.5	198.5	256.3	146.6	256.7	187.1	297.6	187.9	316.1	220.4
BLYP	264.8	222.0	278.7	189.5	224.1	201.3	262.7	147.5	262.0	189.1	305.4	189.3	323.3	222.1
B3LYP	236.9	197.2	240.0	172.1	193.6	182.4	225.6	135.5	233.0	167.1	265.1	173.8	282.1	204.0
BHandHLYP	204.6	174.7	202.4	166.9	167.5	159.2	189.8	127.8	198.4	141.9	227.2	161.4	240.9	188.1
BHandHLYP^[b]^	182.3	165.8	189.8	159.5	152.2	147.7	176.7	122.6	177.1	135.1	210.6	153.5	223.9	179.9
BHandHLYP^[c]^	211.2	176.3	197.4	162.1	170.1	156.2	186.4	124.6	204.7	139.0	224.0	157.0	237.2	182.1
*Periodic model* ^[d]^														
PBE^[e]^	239.7	213.1	428.8	226.8	198.7	162.1	343.8	166.0	193.2	156.9	436.1	194.5	467.0	225.7
PBE	270.6	226.7	295.2	216.0	229.2	224.8	271.4	163.4	272.9	192.4	314.1	203.7	337.2	242.9
BLYP^[f]^	275.5	228.9	301.6	217.0	236.7	227.6	277.7	164.3	277.9	194.4	321.7	205.1	344.2	244.5
B3LYP^[f]^	247.3	203.4	262.3	199.3	205.7	208.3	240.0	152.0	248.6	171.8	280.7	189.4	302.3	226.1
BHandHLYP^[f]^	214.2	180.2	223.8	194.1	179.1	184.5	203.3	144.2	213.1	145.9	241.9	176.7	260.1	209.9
BHandHLYP^[g]^	229.3	187.3	229.7	200.4					227.4	152.3	250.2	191.2		
BHandHLYP^[h]^	235.9	188.9	224.7	195.6					233.8	149.4	247.0	186.7		
Experimental	228.5	183.3												

[a] Scalar relativistic SR‐ZORA calculations of a cluster model of one clathrate cavity with ADF code^73^, ^74^ using Xe/other=jcpl/TZP basis sets.^76^ ^129^Xe chemical shift (δ=σ_Xe−atom_−σ) with respect to free Xe atom shielding values (σ_Xe−atom_): PBE/BLYP/B3LYP/BHandHLYP=5752.2/5752.6/5752.1/5752.0 ppm. Δδ (CSA) defined in principal axis system (PAS) along the unique axis (perpendicular to the plane of O−H rings). Asymmetry parameter η=0 in all cavities due to the cylindrical symmetry. [b] As footnote [a], but at nonrelativistic (NR) level of theory with σ_Xe−atom_(BHandHLYP)=5643.4 ppm. [c] As footnote [a], but at relativistic spin‐orbit SO‐ZORA level of theory with σ_Xe−atom_(BHandHLYP)=6609.3 ppm. [d] Scalar relativistic GIPAW results obtained with CASTEP code.^71^, ^72^ All periodic ^129^Xe chemical shifts referenced to CASTEP/PBE value: σ_Xe−atom_=5926.3 ppm. [e] In the CSP optimized crystal geometry. [f] As footnote [d], but estimated by scaling GIPAW/PBE PAS components with factors obtained at SR‐ZORA level for cluster model resulting in data in footnote [a]. [g] Correction added to result of footnote [f] due to thermal averaging (AVE) over Xe motion at *T=*300 K by MC‐NVT simulation (see text for details). [h] Relativistic spin‐orbit (SO) corrections obtained as difference of SO‐ZORA and SR‐ZORA calculations with ADF code added to result of footnote [g].

**Figure 8:**
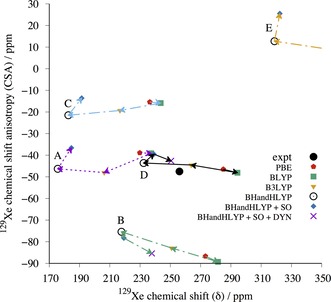
The periodic GIPAW results for the five most likely *o*‐fluorophenol structures optimized at the periodic PBE‐TS level of theory. The labels A–E correspond to structures *o*F_A to *o*F_E in the text. The correct structure is expected to approach the experimental (expt) ^129^Xe NMR parameters, when the computed GIPAW/PBE result is scaled with factors obtained using different pure and hybrid DFT functionals with increasing amount of exact exchange in the series of PBE→BLYP(0 %)→B3LYP(20 %)→BHandHLYP(50 %). The SO correction is added to BHandHLYP values of all structures (blue diamonds). For structures *o*F_B and *o*F_D, the final points (indigo crosses) include also the effect of Xe dynamics (DYN) at *T=*300 K.

**Figure 9:**
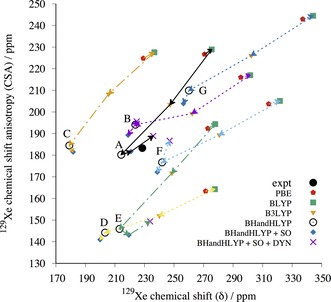
The periodic GIPAW results for the seven *m*‐fluorophenol structures optimized at the periodic PBE‐TS level of theory. The labels A–G correspond to structures *m*F_A to *m*F_G in the text. The correct structure is expected to approach the experimental (expt) ^129^Xe NMR parameters, when the computed GIPAW/PBE result is scaled with factors obtained using different pure and hybrid DFT functionals with increasing amount of exact exchange in the series of PBE→BLYP(0 %)→B3LYP(20 %)→BHandHLYP(50 %). The SO correction is added to BHandHLYP values of all structures (blue diamonds). For structures *m*F_A, *m*F_B, *m*F_E, and *m*F_F, the final point (indigo crosses) include also the effect of Xe dynamics (DYN) at *T=*300 K.

The ADF/SR‐ZORA results for PBE‐TS optimized cluster models were used to extract correction factors (see details in Supporting Information) by which the periodic, SR GIPAW/PBE results were scaled in order to obtain periodic estimates of ^129^Xe NMR parameters with BLYP, B3LYP, and BHandHLYP functionals. The estimated periodic data for the five and seven candidates of *o*‐fluorophenol and *m*‐fluorophenol clathrates are listed in Tables 1 and 2 as well as displayed in Figure 8 and 9, respectively. The best BHandHLYP‐scaled periodic estimates bring one clathrate structure for each of the fluorophenol isomers into excellent agreement with the experimental NMR: *o*F_D and *m*F_A. For *o*‐fluorophenol, the other CSP candidates in the PBE‐TS crystal geometry have quite different periodic BHandHLYP ^129^Xe CS and/or CSA values as compared to the experimental ones, whereas for *m*‐fluorophenol the closest alternative candidates overestimate either the CS (*m*F_F) or CSA (*m*F_B). The BHandHLYP functional is expected to provide reasonable approximation for both quantities due to benchmarking against ab initio calculations.^61^, ^62^, ^63^

In addition, the relativistic SO correction, obtained as the difference between SO‐ and SR‐ZORA cluster calculations with ADF (see Tables 1 and 2), is added to the static BHandHLYP results of all structures (Figure 8 and Figure 9). Due to its different physical origin, the SO effect is case‐specific and may either increase or decrease the CS and CSA, although the latter is affected slightly more. The magnitude of the SO correction is, however, smaller than the dynamical correction (see below) and, hence, does not alter the identification of the best clathrate candidates.

The temperature effect of Xe dynamics at *T=*300 K was modeled for the two (*o*F_B and *o*F_D) and four (*m*F_A, *m*F_B, *m*F_E, and *m*F_F) most relevant clathrate candidates. The dynamical (DYN) correction was added to the SO corrected BHandHLYP results (BHandHLYP+SO). In all cases, the thermal averaging increases the magnitudes of both CS and CSA of ^129^Xe. As seen in Figures 8 and 9, the inclusion of DYN correction confirms the most probable clathrate structure of both isomers. The effect on the simulated spectra is also shown in Figure 2.

It is evident from Figure 8 and 9 (see also Tables S6 and S7 in the Supporting Information) that in all probability the crystal structures for *o*‐ and *m*‐fluorophenol clathrates are *o*F_D and *m*F_A. For them, the ^129^Xe NMR isotropic chemical shift and chemical shift anisotropies at different levels of theory encompass the experimental data. For both structures, the experimental NMR parameters are approached as the computational level is improved. The approximated periodic BHandHLYP results at PBE‐TS optimized structures only slightly underestimate the chemical shift, leaving room for improvements by dynamic and relativistic SO corrections. Astonishingly, the most accurate level of theoretical modeling almost quantitatively reproduces the two NMR parameters that are clearly specific for a given clathrate. The remaining differences between experimental and computational data may be attributed to deficiencies in the structure as well as treatments of electron correlation and thermal averaging of the whole system.

The PXRD results provide further evidence for the CSP‐^129^Xe NMR determined structure of the *o*‐fluorophenol clathrate; the simulated diffraction pattern from *o*F_D is similar to the PXRD obtained from the clathrate sample (see Figure S11 in Supporting Information). PXRD of the present quality cannot, however, be used to unambiquously distinquish between the candidates structures.

### Description of the proposed clathrate structures

**Figure 10:**
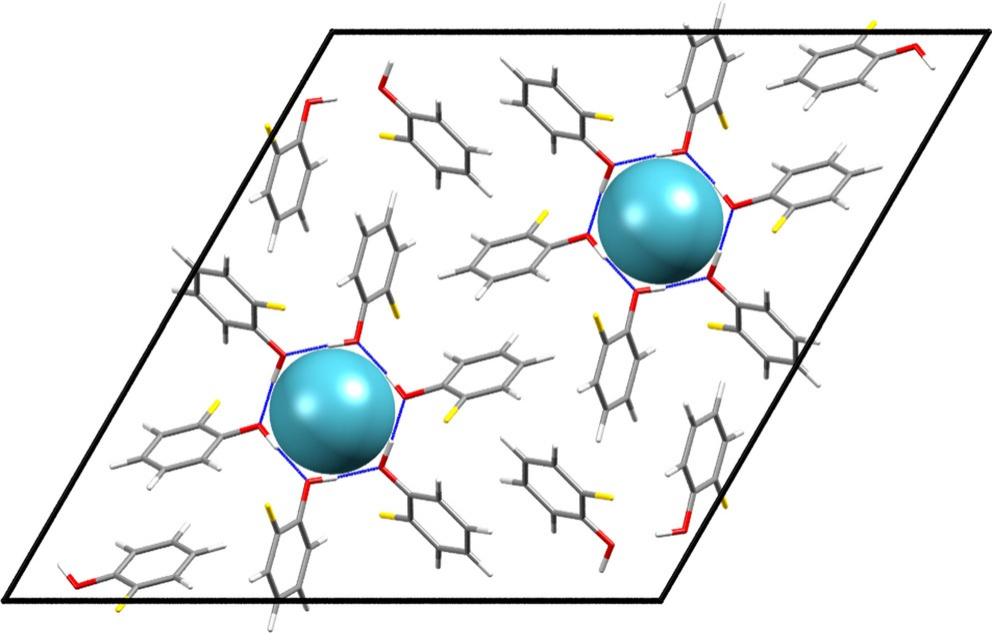
Packing diagram of the PBE‐TS optimized *o*‐fluorophenol xenon clathrate structure *o*F_D viewed down the *c*‐axis. Space group *R* $\bar{3}$ and with R66 (12) hydrogen bonds encasing the xenon atoms. See also Figure 5 a.

**Figure 11:**
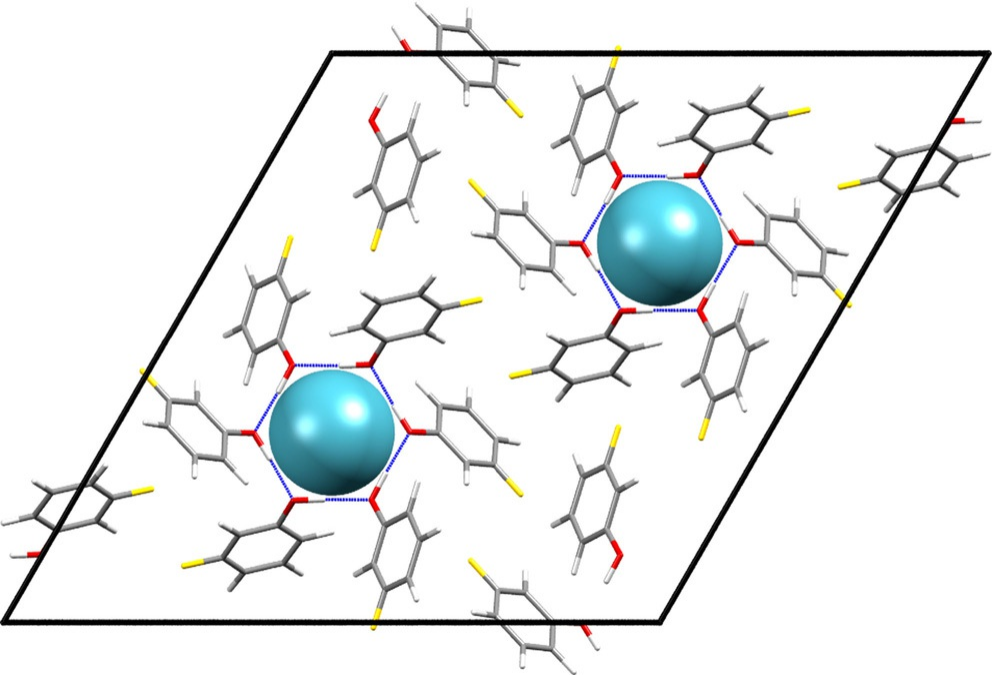
Packing diagram of the PBE‐TS optimized *m*‐fluorophenol xenon clathrate *m*F_A viewed down the *c*‐axis. Space group *R* $\bar{3}$ and with R66 (12) hydrogen bonds encasing the xenon atoms. See also Figure 5 b.

The crystal structures that were proposed as the fluorophenol xenon clathrates belong to space group *R* $\bar{3}$ , with lattice parameters shown in Table 3. Both structures have three‐fold screw axes parallel to the ***c*** lattice vector, resulting in six‐membered rings of host molecules held together with strong hydrogen bonds formed by practically ideal OH⋅⋅⋅O interactions. The hydrogen bonding forms a R66 (12) graph set,^84^ which seems to be a characteristic feature in clathrates of phenol derivatives. Packing diagrams are displayed in Figures 10 and Figure 11. Crystallographic information files (CIFs) are included in the Supporting Information. Both structures have three cavities per unit cell, with volumes 61.4 and 88.5 Å^3^ each for *o*‐ and *m*‐fluorophenol, respectively, resulting in *R_g_* values of 68.7 and 47.7 % for xenon, both within 1.5 standard deviations from the ideal ratio of 59 %.

**Table 3:** Unit cell parameters (after PBE‐TS geometry optimization) for the proposed xenon clathrate structures of *o*‐ and *m*‐fluorophenol.

Structure	space group	*a* [Å]	*b* [Å]	*c* [Å]	*γ*[°]
*o*F_D	*R* $\bar{3}$	22.5225	22.5225	5.6879	120
*m*F_A	*R* $\bar{3}$	22.5526	22.5526	5.6998	120

Despite the same hydrogen bonding and crystal symmetry, the voids in the two structures are of quite different geometry. The void in *o*F_D has an oblate shape with ratios of the principal moments of the free volume (calculated from a grid sampling in Platon)^85^ of 1.00:1.00:0.85, with the short dimension oriented along the crystallographic *c*‐axis. In contrast, *m*F_A has a prolate shape with calculated ratio of dimensions of 1.00:1.00:1.20, elongated along the crystallographic *c*‐axis. Thus, the sign of the CSA relates to the shape of the void, as expected, with an oblate cavity leading to a negative CSA and prolate cavity yielding a positive CSA.

The proposed structure of the *o*‐fluorophenol Xe clathrate (*o*F_D) is one of the highest energy and one of the densest structures that was considered as a possible clathrate (Figure 3). The stabilization of this structure must derive from the interactions of Xe with the host structure, which should be large, due to the tight fit of Xe to the host cavities.

In contrast, the most likely structure of the *m*‐fluorophenol Xe clathrate (*m*F_A) corresponds to the most stable structure on the crystal structure landscape (Figure 4) that contains cavities suitable for Xe enclathration; this structure's stability relates, in part, to the stability of the host framework.

## Conclusion

Detailed first principles NMR calculations have been used on candidates from CSP to propose structures for the xenon clathrates of *o*‐ and *m*‐fluorophenol. The exceptional sensitivity of the ^129^Xe chemical shift tensor to its local environment allows comparisons between observed and calculated NMR chemical shift parameters that have been used to directly confirm or reject hypothetical clathrate structures. Based on these comparisons, likely crystal structures were proposed for the two clathrates. The proposed structures strongly resemble a previously known β‐hydroquinone xenon clathrate^6^ and have similar R66 (12) hydrogen bonding motifs.^84^

The unusual *o*‐fluorophenol ^129^Xe NMR powder spectrum, with its negative CSA, was initially thought to suggest a structural motif different from the known and common R66 (12) hydrogen‐bonded double sandwich. Our results, however, confirm that the R66 (12) motif is an important feature for clathrates of phenol derivatives that is present in both materials studied here.

Static solid‐state NMR spectroscopy has clear benefits over high‐frequency magic‐angle spinning NMR spectroscopy, that would only result in isotropic chemical shifts rather than the complete chemical shift tensors. In this study, using isotropic shifts only would not have allowed the identification of the experimental clathrate structures among the predicted candidates.

The method presented here is a powerful approach for structure determination of porous materials, which is particularly useful in cases where powder diffraction patterns either cannot be obtained, or are insufficient for structure determination. The success of the method is related to the high sensitivity of the ^129^Xe NMR chemical shift and chemical shift anisotropy to minute details of the cavity geometry, such that even structurally very similar clathrates can have vastly different chemical shift tensors. This sensitivity, however, also requires highly detailed NMR calculations; in addition to the proper treatment of electron correlation and relativistic phenomena, the inclusion of explicit crystal lattice effects, as well as Xe dynamics, was necessary in order to precisely reproduce experimental NMR values. Developments in this area, along with progress in CSP algorithms, open up new possibilities for the prediction and characterization of new porous materials by combining structure prediction with computational and experimental ^129^Xe NMR spectroscopy.

## Supporting information

As a service to our authors and readers, this journal provides supporting information supplied by the authors. Such materials are peer reviewed and may be re‐organized for online delivery, but are not copy‐edited or typeset. Technical support issues arising from supporting information (other than missing files) should be addressed to the authors.

Supplementary
