# Supplementary material for: Clathrate Structure Determination by Combining Crystal Structure Prediction with Computational and Experimental 129Xe NMR Spectroscopy
Source: Chemistry. 2017 Feb 14;23(22):5258–69. doi: 10.1002/chem.201604797 (PMC5763392; doi:10.1002/chem.201604797)
Supplement: Supplementary file 1 — Supplementary [file CHEM-23-5258-s001.pdf]

CHEMISTRY
A European Journal

Supporting Information

Clathrate Structure Determination by Combining Crystal Structure Prediction with Computational and Experimental ^{129}Xe NMR Spectroscopy

Marcin Selent+,[a, c] Jonas Nyman+,[b] Juho Roukala,[a] Marek Ilczyszyn,[c] Raija Oilunkaniemi,[d] Peter J. Bygrave,[b] Risto Laitinen,[d] Jukka Jokisaari,[a] Graeme M. Day,*[b] and Perttu Lantto*[a]

chem_201604797_sm_miscellaneous_information.pdf

The following structure files, in CIF format, accompany this document:

mF_all.cif and oF_all.cif: all predicted crystal structures of both molecules, in order of increasing energy.

mF_hosts.cif and oF_hosts.cif: all predicted crystal structures with voids suitable for hosting a xenon atom (see below for explanation).

mF_clathrates.cif and oF_clathrates.cif: all selected host crystal structures after computational insertion of a xenon atom, followed by lattice energy minimisation (see below for explanation).

mF_A_PBE-TS.cif, mF_B_PBE-TS.cif, mF_C_PBE-TS.cif, mF_D_PBE-TS.cif, mF_E_PBE-TS.cif, mF_F_PBE-TS.cif, and mF_G_PBE-TS.cif. Periodic DFT optimised crystal structures of the 7 predicted clathrate structures of *m*-fluorophenol with NMR parameters closest to the experimentally measured parameters.

oF_A_PBE-TS.cif, oF_B_PBE-TS.cif, oF_C_PBE-TS.cif, oF_D_PBE-TS.cif, and oF_E_PBE-TS.cif. Periodic DFT optimised crystal structures of the 5 predicted clathrate structures of *o*-fluorophenol with NMR parameters closest to the experimentally measured parameters.

1 Crystal structure prediction (CSP)

The CSP calculations involve five steps: i) the lowest energy conformations of the isolated molecules are identified; ii) hypothetical crystal packings are generated and the lattice energy is minimised using a simple force field and rigid-molecule constraints; iii) a subset of the lowest energy predicted crystal structures are re-optimised using an anisotropic atom-atom force field; iv) intramolecular flexibility is introduced, allowing the hydroxyl group to reorient in response to packing forces in each low energy crystal structure; v) porous structures are identified, xenon atoms are inserted into the pores and the structures once again re-optimised.

1.1 Molecular conformational analysis

Both molecules have rigid molecular geometries, apart from the orientation of the hydroxyl group. The two compounds *o*-fluorophenol and *m*-fluorophenol both have a single dihedral angle (H-O-C-C) and the dihedral angle is likely to have a large impact on the possible crystal structures and their lattice energy. The dihedral angles of both molecules were examined by scanning them in steps of 5° and each conformation was geometry optimised using Gaussian09 version D01 and the B3LYP hybrid functional and Møller-Plesset second order perturbation theory. The *cis*/0° and *trans*/180° conformers, where the -OH hydrogen is pointing towards the fluorine, and away from it, respectively, have the lowest energy. In *o*-fluorophenol the difference in energy between the *cis* and *trans* conformers is 13 kJ/mol and in *m*-fluorophenol, it is only 0.1 kJ/mol.

1.2 Crystal structure generation

A large set of hypothetical crystal structures was generated with the rigid DFT-optimised molecular geometries using Monte Carlo simulated annealing[1] with the Polymorph Predic-

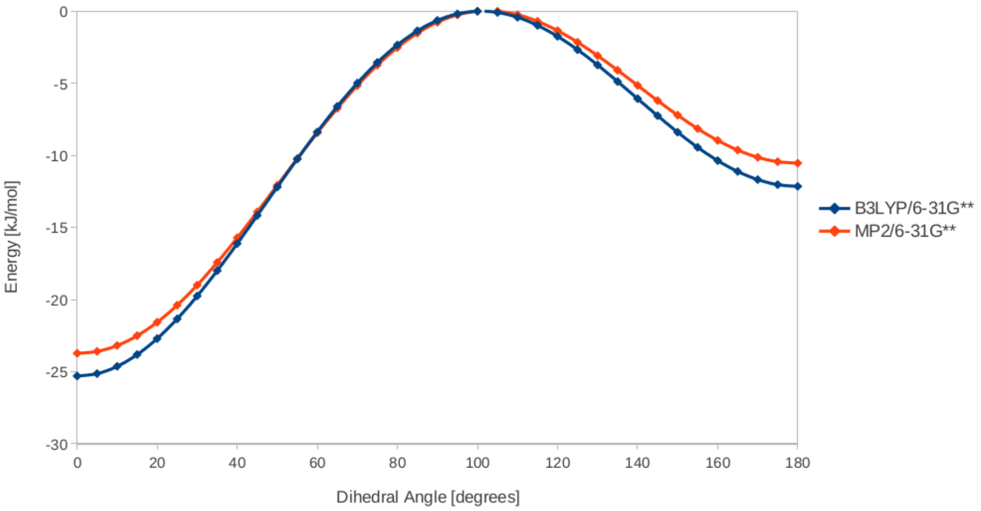

Figure S1: *o*-fluorophenol dihedral angle scan.

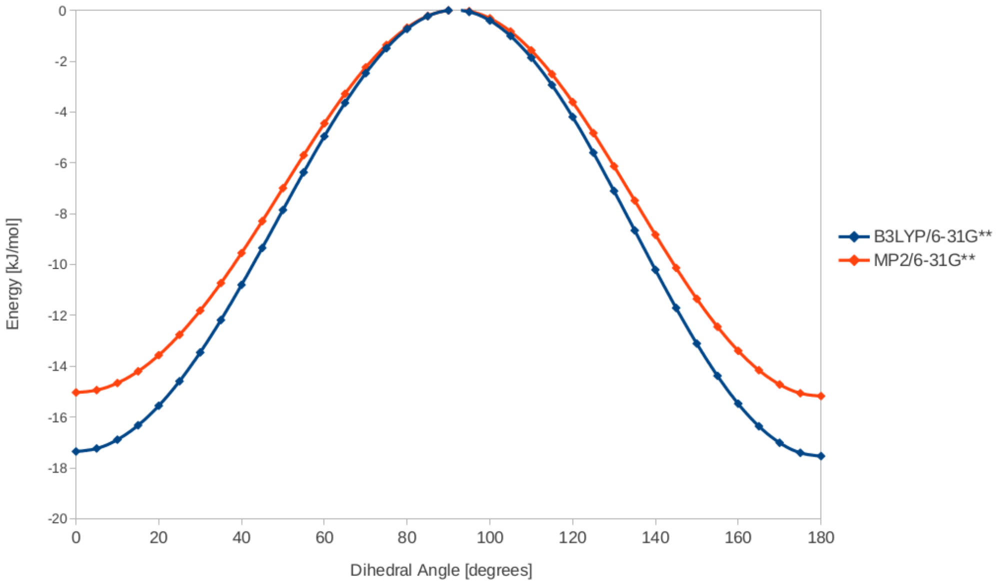

Figure S2: *m*-fluorophenol dihedral angle scan.

TOR module in Accelrys' MATERIALS STUDIO software. Searches were performed using space group symmetry in the most commonly observed space groups of known crystal structures in the Cambridge Structure Database (CSD). There are reasons to believe that clathrate structures occur selectively in certain space groups[2,3], but no space groups were omitted because of this. Searches with one molecule in the asymmetric unit ($Z' = 1$) were performed in 25 space groups ($P2_1/c$, $P2_12_12_1$, $P\bar{1}$, $P2_1$, $Pbca$, $C2/c$, $Pna2_1$, Cc, $C2$, $Pca2_1$, $P1$, $Pbcn$, $P4_12_12$, $P2_12_12$, Pc, $P4_1$, $Fdd2$, $Pccn$, $P2/c$, $P6_1$, $I4_1/a$, $P3_2$, $R3$, $R\bar{3}$, $C222_1$) and were repeated for both planar conformers of both molecules. $Z' = 2$ searches were performed in $P1$, $P\bar{1}$, $P2_1$, $P2_1/c$ and $P2_12_12_1$ with the three possible combinations of conformers for each molecule. Ten simulated annealing cycles starting from different random seeds were deemed to sample the search space adequately for $Z' = 1$, since most lowest energy structures in each space group had been located multiple times. For $Z' = 2$, 10–25 repeats were deemed adequate, depending on the space group.

Hypothetical crystal structures were generated by simulated annealing in the Polymorph mod-

ule (version 6.1, Build date March 7, 2013) in Materials Studio version 6.1.200. Several Monte Carlo simulated annealing calculations were performed starting with different random number seeds, until almost all of the 10 lowest energy crystal structures had been found more than once. This meant approximately 10 runs were required for each space group with $Z' = 1$ and 20 runs per space group for $Z' = 2$.

The following settings were used:
Search algorithm : MC Simulated Annealing
Maximum number of steps : 10000
Explore torsions : No
Preoptimize structures : No
Steps to accept before cooling : 14
Minimum move factor : 0.1000E-09
Heating factor : 0.02500
Maximum temperature : 150000.0 K
Minimum temperature : 300.0 K

Intermolecular interactions at this stage were modelled using the COMPASS force field[4] with atomic partial charges fitted to the molecular electrostatic potential using the CHelpG method[5]
Optimisation algorithm : Smart
Convergence tolerances:
Energy : 1.5e-005 kcal/mol
Force : 0.001 kcal/mol/Å
Stress : 0.001 GPa
Displacement : 1e-005 Å
Maximum number of iterations : 500
External pressure : 0 GPa
Motion groups rigid : YES
Optimize cell : YES

Forcefield : COMPASS
Electrostatic terms:
Summation method : Ewald
Accuracy : 1e-05 kcal/mol Buffer width : 0.5 Å

van der Waals terms:
Summation method : Atom based
Truncation method : Cubic spline
Cutoff distance : 18.5 Å
Spline width : 1 Å
Buffer width : 0.5 Å

The generated structures were clustered to remove duplicates. Clustering was performed with COMPACK using these settings:
Cluster grouping : Forcefield type
Cutoff : 7.000 Å

Number of bins : 140
Tolerance : 0.1100

Maximum number of clusters : All clusters

1.3 Lattice energy minimisation

Structures with a lattice energy within a 15 kJ/mol window from the lowest energy structure were further refined with an anisotropic intermolecular potential model. Lattice energies were calculated from a hybrid method, combining intramolecular DFT energies with an intermolecular force field model:

$$E_{\text{latt}} = E_{\text{intra}}^{\text{DFT}} + E_{\text{inter}}^{\text{atom-atom}}. \tag{1}$$

Intramolecular energies and molecular geometries were derived from DFT. Intermolecular interactions were modelled with an anisotropic model potential of the form:

$$E_{\text{inter}}^{MN} = \sum_{i,k} \left(A^{\iota\kappa} \exp\left(-B^{\iota\kappa} r_{ik}\right) - C^{\iota\kappa} r_{ik}^{-6} + E_{ik}^{\text{elec}} \right), \tag{2}$$

where i, k are atoms of types ι and κ in molecules M and N, respectively, separated by the distance r_{ik}. The first two terms describe the repulsive and attractive non-electrostatic intermolecular interactions, with $A^{\iota\kappa}$, $B^{\iota\kappa}$ and $C^{\iota\kappa}$ being empirically determined parameters. The final term, describing electrostatic interactions, was calculated from atom-centered multipoles up to hexadecapole on all atoms, obtained from a distributed multipole analysis[6] of the molecular charge density using GDMA[7]. The multipole model accurately describes anisotropic intermolecular interactions, such as hydrogen bonds and π-stacking. Charge-charge, charge-dipole and dipole-dipole interactions were calculated using Ewald summation, while repulsion-dispersion interactions and all higher multipole-multipole interactions were summed to a cutoff of 15 Å.

A revised version of the W99 exp-6 potential[8] was used, whose re-parametrisation optimised its performance for hydrogen bonding when used together with atomic multipole electrostatics[9]. Fluorine atoms were treated as having an anisotropic repulsion, as described previously[10,11]. All intermolecular interaction parameters are provided below. W99 requires all X-H bonds in the DFT-optimised molecular geometry to be foreshortened by 0.1 Å. Using this model potential, crystal structures were lattice energy minimised with molecular geometries kept rigid, using DMACRYS[10].

Duplicate crystal structures were removed using the COMPACK[12] program, which compares interatomic distances in a cluster of 15 molecules from each crystal structure.

The resulting unique structures were re-optimised using the CRYSTALOPTIMIZER[13] program to treat flexibility of the hydroxyl group. CRYSTALOPTIMIZER minimises the sum of intra- and intermolecular energies by calculating the strain due to crystal packing forces on the hydroxyl dihedral angle and allows this to relax. The minimisation is performed by iteratively computing the intermolecular forces on the flexible dihedral angle in DMACRYS, and optimising the intramolecular geometry in GAUSSIAN under the influence of the packing forces.

CIF-files of all predicted crystal structures are included (mF_all.cif and oF_all.cif). Structures within the CIF are ordered by increasing lattice energy.

2 Crystal structure overlays

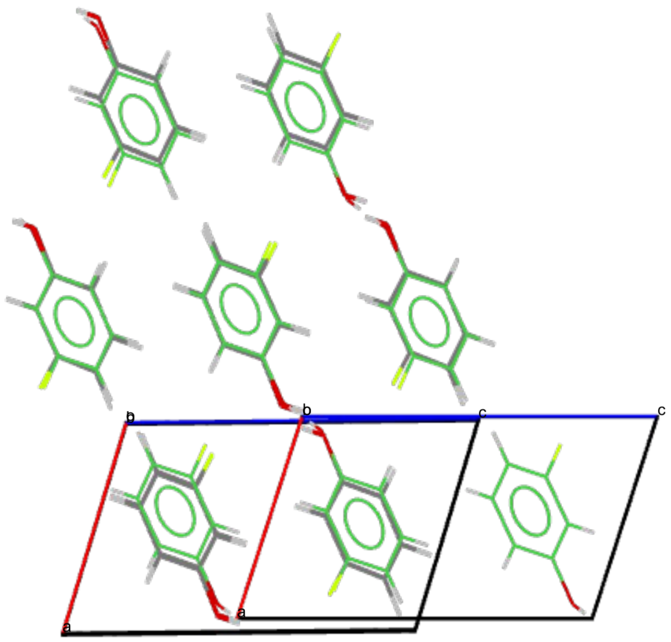

Figure S3: Overlay of the lowest energy predicted *m*-fluorophenol crystal structure mF0_P21_3110 with QAMTUU. The match is excellent with $RMSD_{15} = 0.203$ Å.

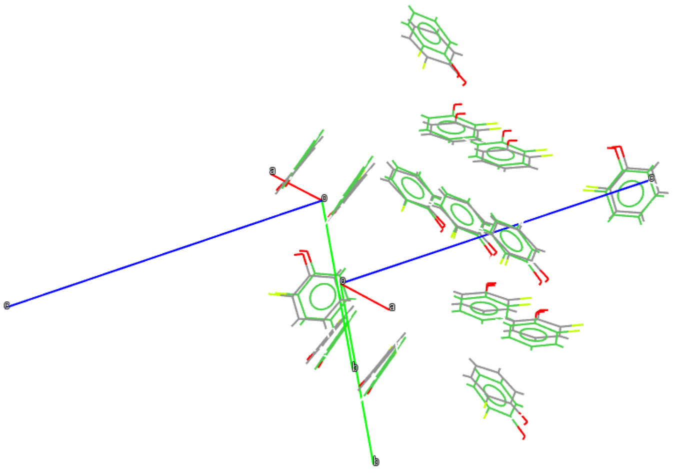

Figure S4: Overlay picture of the predicted structure oF00_P212121_5 with QAMWEH01. The experimental structure was determined at elevated temperature and pressure, making a structure comparison difficult. Re-optimising the predicted structure at the experimental pressure (0.36 GPa) gives a match with $RMSD_{15} = 0.422$ Å.

3 Guest to host volume ratios

Molecular volumes were calculated as the sum of atomic fragment contributions using the Molinspiration property calculator available online[14] and a volume of 42.2 Å^3 was used for xenon. Crystalline cavity volumes were calculated by placing probe particles at positions in a regular grid with 0.2 Å spacing in the unit cell and testing if the particle was within any atom's van der Waals radius,[15,16] as implemented in the program PLATON.[17] Convergence of the calculated void with respect to grid spacing is discussed below. PLATON allows not only the total void volume per unit cell to be determined, but also the volume of each individual pore, and that is the volume used in this work.

Void volumes were calculated with the PLATON program which places a test particle of a certain radius at positions in a regular grid in the unit cell. The calculation is highly sensitive to the particle size and grid spacing. Using a large probe will underestimate the volume and will often result in an erroneous topology of the void space. Small molecules may diffuse through narrow channels between larger cavities. We have chosen a probe size of 1.2 Å radius, corresponding to the kinetic diameter of a H_2 molecule, this is also the default setting in PLATON. A coarse grid will result in inaccurate volumes. A convergence test was carried out on a subset of the known clathrates in CSD.

The relative error of the calculated void volume for different grid spacing was compared to the very fine grid spacing of 0.1 Å.

$$\text{Error} = 100 \cdot \left(\frac{V_{\text{grid}} - V_{0.1}}{V_{0.1}} \right) \tag{3}$$

The computational cost prevents the finest grid spacings to be used routinely. The convergence test indicated that the void volume calculation is slowly converging for grid spacings smaller than 0.4 Å. Grid spacings larger than 0.3 Å produces unacceptably large errors. A grid spacing of 0.2 Å was deemed adequate, as it consistently gives volumes with small errors of approximately 5% relative to the 0.1 grid spacing. By extrapolating the curves in the convergence diagram, the true errors relative to a perfect grid spacing of zero can be estimated. The volumes calculated by a 0.2 Å grid spacing are systematically underestimating the true volume by about 8%.

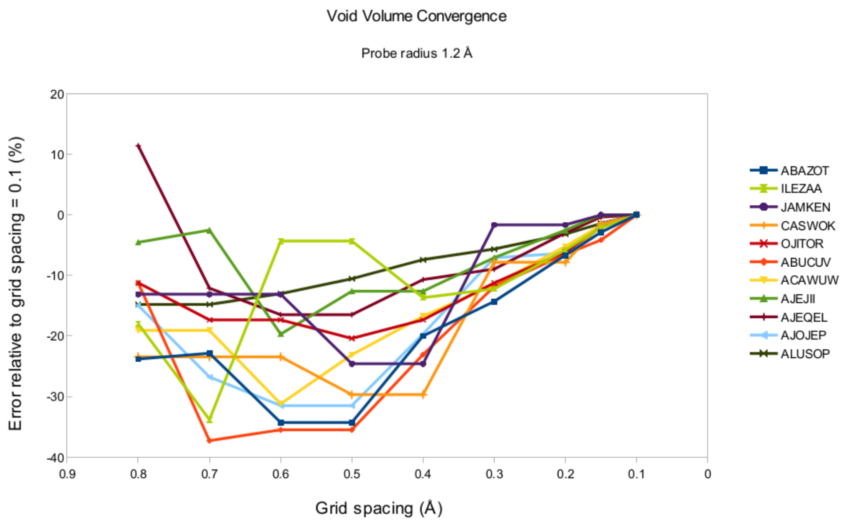

Figure S5: Convergence test for the void calculation

In order to confirm the validity of the 55% rule[18] for our void calculation method, the CSD was searched for organic clathrate structures (see Table S1). Disordered structures, metallo-organic structures, and hydrates were excluded. In addition, the hydroquinone SO_2 and hydroquinone C_{60} clathrates were used. The set of selected structures was chosen only to be large enough to estimate the spread in guest-to-host ratio and to represent a variation in host guest chemistry.

The guest molecules were removed from the structure and a void calculation was performed using a 1.2 Å probe radius and 0.2 Å grid spacing. Guest molecule volumes other than Xe were calculated with the Molinspiration property engine v2013.09, by Molinspiration Cheminformatics 2014. It is available online at `http://www.molinspiration.com/cgi-bin/properties`. It provides volumes calculated from summing atomic group volumes fitted to vacuum AM1 semiempirical conformations of a large training set. Volumes in Å^3. For the volume of xenon, the van der Waals volume 42.2 Å^3 was used.

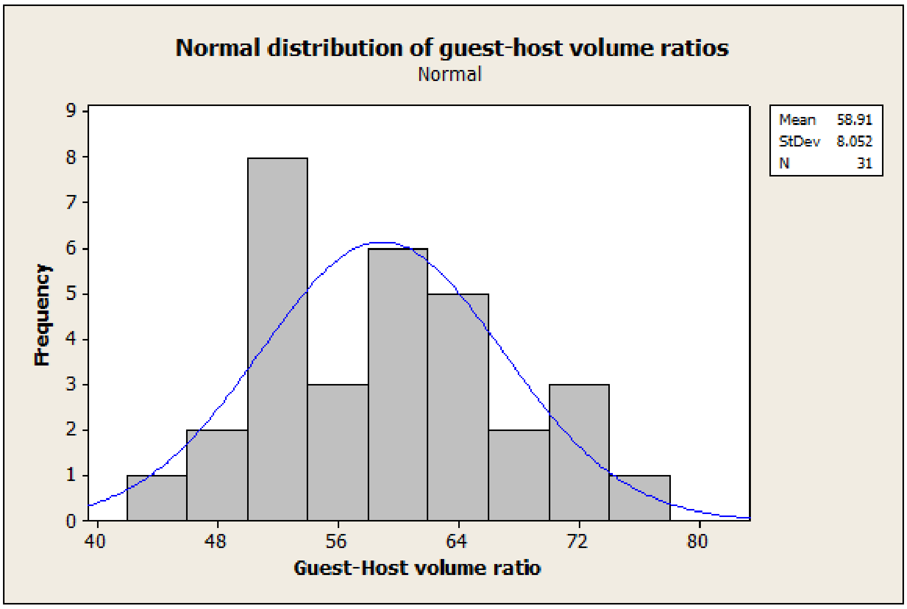

Figure S6: The Guest-to-host volume ratios are normally distributed around 58.9% with a standard deviation of 8.0 percentage-points.

Guest to host volume ratios are normally distributed around 60% with a standard deviation of 8 percentage points (see Figure S6).

CIF-files of selected host structures from the CSP sets of structures are included in oF_hosts.cif and mF_hosts.cif. These structures, after insertion of Xe and re-optimisation, are included in oF_clathrates.cif and mF_clathrates.cif.

CSD code	# of guests	Guest	Guest Vol	Void Vol	Ratio %
ABAZOT	2	3-pentanone	98.344	327	60.14
ABUCUV	2	acetonitrile	46.055	158	58.28
ACAWUW	2	ethanol	54.016	164	65.86
AJEJII	3	acetonitrile	46.055	193	71.58
AJEQEL	4	chloroform	70.07	553	50.68
AJOJEP	1	chloroform	70.07	119	58.88
ALUSOP	2	chloroform	70.07	275	50.96
ANIRUL	1	butan-1-ol	87.619	171	51.23
ASOKOI	1	TCE	100.434	154	65.21
AVESOJ	4	diethylamine	91.763	694	52.88
BABYIN	2	acetonitrile	46.055	171	53.86
BAFSOQ	2	urea	54.196	154	70.38
BAZJES	1	acetone	64.74	122	53.06
BEGZAP	2	propionic acid	72.999	285	51.22
BERYED	4	methane	28.644	193	59.36
BICYCLO	2	diethylether	88.345	271	65.18
BIFQAI01	2	benzonitrile	100.903	408	49.46
BIJVOf	3	acetid acid	46.055	217	63.66
BOSVEK01	1	sulphur dioxide	41.314	61	67.72
BUSPAG	1	methylisocyanide	51.287	79	64.92
CAKGUQ	1	acetonitrile	46.055	85	54.18
CAVMES	1	diethylether	88.345	271	65.18
GAGNEJ	2	DMSO	71.433	216	66.14
GIRBOY	3	xenon	42.2	226	56.01
HQ-C60	2	C60	539.5	1945	55.46
HQUACN	1	acetonitrile	46.055	77	59.81
HQ β	1	sulphur dioxide	41.314	68	60.75
ILEZAA	1	xenon	42.2	84	50.23
JAMKEN	1	xenon	42.2	60	70.33
LOZNUJ	1	xenon	42.2	55	76.72
OJITOR	1	xenon	42.2	92	45.86

Table S1: Structures and void properties used in determining selection rules for inclusion hosts. In summary: 58.9 $\pm$ 8.0 (1 σ). TCE = 1,1,2,2-tetrachloroethane

4 Revised Williams99 parameters

All parameters describing interactions between C, N, O and H atoms are described using Williams99[8] forcefield parameters, apart from hydrogen bond H...A interactions, which have been re-parameterised[9] to work more effectively with the atomic multipole electrostatic model. For H...A interactions, the pre-exponential parameter of the exp-6 model was modified from the Williams99 value. The parameters are given in the following table.

Table S2: Revised H...A parameters in the exp-6 intermolecular model used. C = 0 for all interactions.

hydrogen	acceptor	A (eV)
H2	N1	149
H2	N2	166
H2	N3	163
H2	O1	129
H2	O2	105
H3	N1	70
H3	N2	118
H3	O1	127
H3	O2	133
H4	N1	141
H4	N2	77
H4	N3	56
H4	N4	112
H4	O1	34
H4	O2	198

5 Potential parameters for fluorine

Halogen atoms tend to have an anisotropic van der Waals radius[19]. To account for this, intermolecular potentials with an anisotropic repulsion term have been developed[10,11]. A local unit vector $\mathbf{e}_z$ is defined at each anisotropic site, parallel to the covalent bond joining the halogen to its bonded atom, pointing away from the bond. A second unit vector, $\mathbf{e}_{ik}$, is the vector between the interacting atoms. DMACRYS describes repulsion anisotropy using a modified exp-6 potential of the form:

$$V = G \exp\left(-B^{\iota\kappa}(r_{ij} - \rho^{\iota\kappa}(\Omega_{ik}))\right) - C^{\iota\kappa}/r^6, \tag{4}$$

where $\rho^{\iota\kappa}(\Omega_{ik})$ describes the anisotropy of repulsion, and is defined as:

$$\rho^{\iota\kappa}(\Omega_{ik}) = \rho_0^{\iota\kappa} + \rho_1^{\iota}(\mathbf{e}_z^i \cdot \mathbf{e}_{ik}) + \rho_1^{\kappa}(-\mathbf{e}_z^k \cdot \mathbf{e}_{ik}) + \rho_2^{\iota}(3[\mathbf{e}_z^i \cdot \mathbf{e}_{ik}]^2 - 1)/2 + \rho_2^{\kappa}(3[\mathbf{e}_z^k \cdot \mathbf{e}_{ik}]^2 - 1)/2 \tag{5}$$

ρ_0 describes the isotropic repulsion, ρ_1 parameters describe a shift of the centre of repulsion and ρ_2 parameters describe a quadrupolar distortion of the atom. Parameters for fluorine were taken from Day's specifically developed potential for molecule XIII in the 4th blind test of crystal structure prediction[20]. The parameters were empirically fitted to reproduce the crystal structures of a set of halogenated aromatic molecules. Details are available in the ESI to the 4th blind test paper. The parameters, in input format for DMACRYS are provided below:

```
BUCK   F_01  F_01
   3761.006673    0.240385    7.144500   0.0  70.0
ANIS   F_01  F_01
 0  0  2  0  2 -0.035000
 0  0  0  2  2 -0.035000
ENDS
```

6 Potential parameters for xenon

An exp-6 potential for xenon was parametrised to be used together with the Williams99 force-field[8]. The parameters were fitted to reproduce the potential energy surfaces of van der Waals dimers of $Xe\cdots Xe$[21], $Xe\cdots H_2O$[22], $Xe\cdots CH_4$[23], $Xe\cdots N_2$[24], $Xe\cdots O_2$[25] and $Xe\cdots C_6H_6$[26].

Table S3: Xenon potential parameters

Coefficient	Fitted value	
A	8216.849	eV
B	0.348606	$Å^{-1}$
C	339.32	eV/$Å^6$

Cross terms for heteroatomic interactions are obtained as:

$$A_{ij} = \sqrt{A_i A_j} \tag{6}$$

$$B_{ij} = 0.5 \cdot (A_i + A_j) \tag{7}$$

$$C_{ij} = \sqrt{C_i C_j} \tag{8}$$

7 NMR calculations

7.1 Screening calculations on cluster models

From each predicted clathrate structure, we constructed a cluster model containing a single xenon-occupied cavity. Adjacent fluorophenol molecules with any atoms within 5.3 Å of the Xe atom were included in each cluster. Symmetries of the clusters were not used during geometry optimizations and NMR calculations.

Calculations were performed using the BHandHLYP [27,28] hybrid functional using TURBOMOLE [29]. The MHA basis set [$22s17p14d2f/15s13p11d2f$] was used for Xe, which has been tested and found well-converged for nonrelativistic Xe NMR shielding. [30–32] For other atoms (F, C, O, H) def2-SVP basis sets [33] were used; this basis set combination is denoted MHA/SVP. The MHA/SVP basis set combination with the BP86 [27,34] functional was used in steepest descent DFT optimizations of hydrogen atom positions with fixed heavy atom coordinates. The influence of optimizing the hydrogen positions was examined and found not to be significant for the calculated NMR results (see below), since the clusters were derived from DFT-optimised molecules with already accurate hydrogen positions.

7.2 Periodic modelling of likely candidates

Five *o*-fluorophenol and seven *m*-fluorophenols crystal structures with screening level NMR parameters close to the experimental values were chosen for further scrutiny. These structures were optimised with respect to both atomic positions and lattice parameters using planewave, periodic DFT with the PBE functional [35] and Tkatchenko-Scheffler (TS) dispersion correction [36] using CASTEP [37,38] and denoted as PBE-TS structures from now on. Optimizations were carried out using ultrasoft pseudopotentials [39] with 380 eV cutoff and Γ-point centered Monkhorst-Pack [40] **k**-point meshes with a spacing of ≤ 0.04 Å^{-1}. Convergence thresholds were 10^{-2} eV/Å and $5 \cdot 10^{-4}$ Å for the ionic residual forces and displacements, respectively.

Following optimization, NMR shielding tensors of these PBE-TS optimised models were computed with the PBE functional using the gauge-including projector augmented wave (GIPAW) method [41,42] (see Tables S6 and S7 below). Norm-conserving pseudopotentials [43] were used with a 610 eV cutoff and a more stringent **k**-point spacing of ≤ 0.03 Å^{-1} in all directions, in order to obtain full shielding tensors accurately. These periodic CASTEP results include the scalar relativistic effects on Xe shielding at the 1-component scalar-relativistic (SR) zeroth-order regular approximation [44,45] (SR-ZORA) level of theory.

CIF-files of PBE-TS optimized structures are included (oF_A-E_PBE-TS_structure.cif and mF_A-G_PBE-TS_structure.cif).

The structure of the selected clathrate of o-fluorophenol (oF_D) has space group symmetry $R3$ in the CIF file. This is lower symmetry than the symmetry of the host framework ($R\overline{3}$) due to the xenon atom optimising to a position just away from the centre of symmetry. Thermal motion will average over the positions of the xenon atom, giving the $R\overline{3}$ structure.

7.3 Detailed modelling of the most probable structures.

Detailed calculations were performed on clusters comprising of a single cavity, whose geometries were taken from the periodic, PBE-TS planewave optimizations. Calculations were performed using the Amsterdam Density Functional[46,47] (ADF) code including 1-component scalar-relativistic (SR-ZORA) and 2-component spin–orbit (SO-ZORA) methods.[44,48] The ADF calculations of NMR shielding tensors[45,49,50] employed the finite Gaussian nuclear model[51] with all-electron jcpl and TZP basis sets[52] for Xe and other atoms, respectively.

The influence of the DFT functional and the amount of the exact Hartree-Fock exchange (EEX) was tested by carrying out cluster calculations with pure PBE and BLYP[27,53] (EEX=0%) as well as hybrid B3LYP(EEX=20%)[54,55] and BHandHLYP(EEX=50%) functionals. The SR-ZORA *cluster* results obtained with these functionals in *cluster* calculations were then used in scaling of the *periodic* PBE results. Due to cylindrical symmetric cavities of the most probable crystal structures, all Xe shielding tensors are axially symmetric ($\eta \approx 0$) and, hence, the scaling is carried out for the principal axis system (PAS) components, for example:

$$\sigma_{ii}^{periodic}(\mathrm{BHandHLYP}) \approx \frac{\sigma_{ii}^{cluster}(\mathrm{BHandHLYP})}{\sigma_{ii}^{cluster}(\mathrm{PBE})} \times \sigma_{ii}^{periodic}(\mathrm{PBE}) \quad (i = 1, 2, 3), \qquad (9)$$

which are then used to compute Xe chemical shift δ and chemical shift anisotropy $\Delta\delta$.

7.4 Modelling of the effect of Xe dynamics

The effect of Xe dynamics for ^{129}Xe NMR shielding parameters at $T = 300\,\mathrm{K}$ was modelled for the few most probable clathrates by canonical NVT Metropolis Monte Carlo (MC-NVT) of Xe motion on a potential energy surface (PES) inside a fixed cluster cavity with fixed PBE-TS optimised geometry. An in-house simulation program was used, in which the Xe atom moves on a PES that is piecewise-linearly interpolated from a nonuniform 3D grid of energies.[56] For each clathrate, the 3D grid consists of at least 343 displacement of the Xe atom around the center of the cavity. At the largest displacement in every direction the concave PES value is more than 5 kJ/mol (600 K) higher than the lowest value. The BHandHLYP functional with Grimme's D3 dispersion correction[57] was used in TURBOMOLE calculations of the PES. In every 3D grid point also the full ^{129}Xe NMR shielding tensor was computed at the same level. The above-mentioned def2-SVP basis set was used for light elements of the cluster, while for Xe we used an uncontracted ($27s25p21d4f$) completeness-optimised (co-r) all-electron basis set, which provides practically the basis set limit nonrelativistic Xe NMR shielding tensors.[56] ^{129}Xe NMR shielding tensor elements were averaged in MC-NVT simulation of 10^7 steps with a maximum 1D step-size of 0.15 Å that gives standard errors smaller that 0.1 ppm for all tensor components. The temperature effects on Xe CS and CSA were then computed as the difference of the PAS values of the averaged tensor with respect to the reference Xe shielding tensor at the center of the cage. Due to cylindrically symmetric cavities, all the Xe shielding tensors are axially symmetric in their PAS and, hence, definition of CSA along the symmetric axis is unambiguous and the asymmetry parameter is practically $\eta = 0$ for all tensors.

8 Changes in chemical shift of ^{129}Xe NMR with manipulations on cluster structures

Structures obtained in the CSP process include direct insertion of xenon atom in the lattice voids and further optimization of the periodic structures using empirical potentials. For 11 fluorophenol clathrate CSP-structures with lattice energy close to absolute minimum ^{129}Xe NMR chemical shift and chemical shift anisotropy (CSA) have been calculated for structures (1) without optimization after Xe insertion, (2) optimised with Xe position at the force-field level used in CSP procedure refinements, and (3) structures as in previous but position of hydrogen atoms optimised with DFT. In these calculations, the MHA/SVP basis set combination with the BP86[27,34] functional was used in steepest descent optimizations of hydrogen atom positions with other atom coordinates fixed. Results are presented in Figure S7 in arrows connecting results (1) to (3) of the same CSP structure.

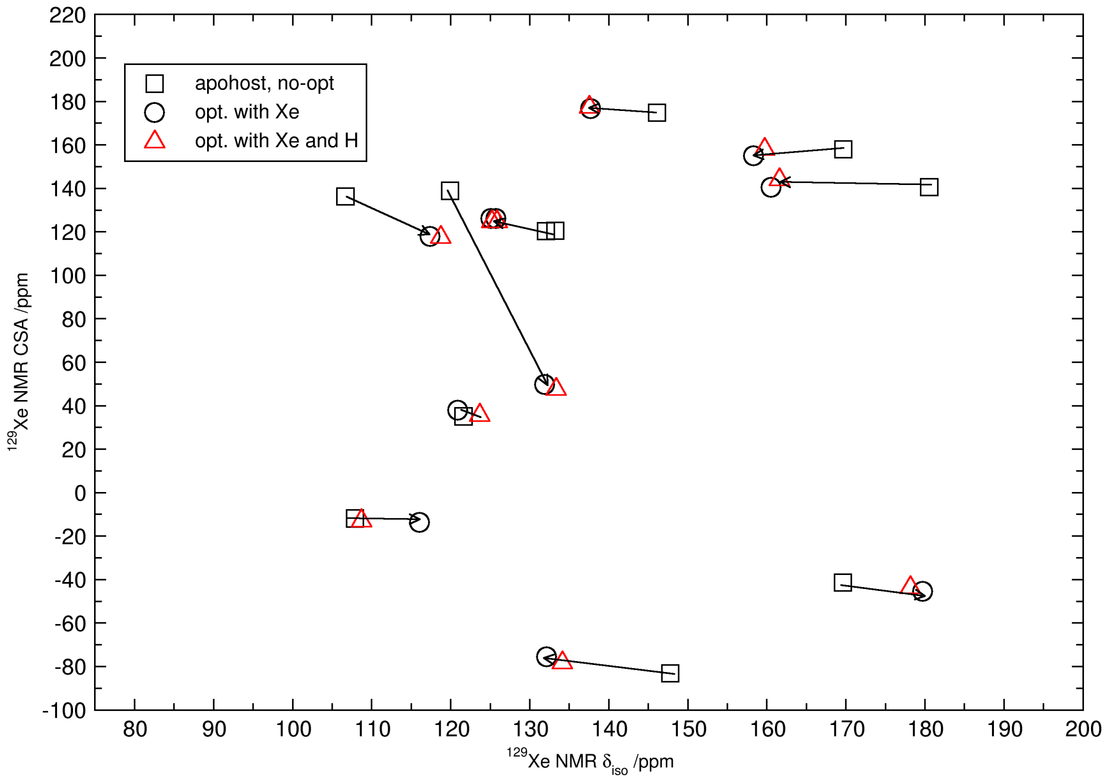

Figure S7: Changes in computed ^{129}Xe NMR parameters due to partial optimization of the CSP clusters.

9 Xe NMR results

In Table S4 we present ^{129}Xe NMR chemical shifts (δ), anisotropies (CSA, $\Delta\delta$) (in ppm) and tensor asymmetries η for 33 CSP-generated *o*-fluorophenol clathrate structures calculated at the screening level of theory.

These calculations were performed on cluster models with nonrelativistic ^{129}Xe NMR shielding tensors computed with TURBOMOLE using the BHandHLYP hybrid DFT functional with the MHA basis set [$22s17p14d2f/15s13p11d2f$] for xenon and def2-SVP basis sets for all other atoms.

Table S4: Calculated chemical shifts and CSAs at the screening level of theory for selected *o*-fluorophenol clathrate structures.

ID	Name	δ	$\Delta\delta$	η
*o*F_A	oF0_R-3_46_Xe	108.7	−12.9	0.0
*o*F_B	oF0_R-3_24_Xe	178.2	−43.6	0.0
*o*F_C	oF0_R-3_20_Xe	147.0	15.2	0.0
*o*F_D	oF180_R-3_51_Xe	181.4	−10.0	0.0
*o*F_E	oF180_R-3_44_Xe	241.4	27.0	0.0
	oF0_R-3_17_Xe	116.2	−20.6	0.0
	oF0_R-3_38_Xe	180.4	167.2	0.0
	oF0_R-3_6_Xe	240.4	67.4	0.0
	oF0_R-3_7_Xe	246.1	200.0	0.0
	oF180_R-3_11_Xe	291.4	39.7	0.0
	oF180_R-3_19_Xe	304.5	25.3	0.0
	oF180_R-3_1_Xe	286.2	196.8	0.0
	oF180_R-3_2_Xe	131.7	31.8	0.0
	oF180_R-3_3_Xe	286.8	31.2	0.0
	oF180_R-3_9_Xe	226.6	188.1	0.0
	oF0_R-3_10_Xe	137.6	177.3	0.0
	oF0_11591_Xe	133.3	47.6	0.0
	oF0_R-3_14_Xe	125.9	124.6	0.0
	oF180_R-3_15_Xe	161.6	143.9	0.0
	oF0_1258_Xe	430.3	−148.8	0.1
	oF0_R-3_39_Xe	72.8	−0.4	0.4
	oF0_13826_Xe	265.1	69.2	0.6
	oF0_5586_Xe	264.4	69.4	0.6
	oF00_P-1_14655_Xe	223.8	−140.9	0.4
	oF00_P-1_14867_Xe	83.6	−37.4	0.4
	oF00_P-1_1752_Xe	77.8	−88.4	0.4
	oF00_P-1_1773_Xe	126.8	−36.2	0.6
	oF00_P-1_1990_Xe	149.8	−144.8	0.7
	oF00_P-1_25199_Xe	62.0	35.8	0.5
	oF00_P-1_2991_Xe	87.7	−28.4	0.8
	oF00_P-1_7665_Xe	98.6	57.7	0.9
	oF00_P21-C_3118_Xe	205.0	−122.0	0.7
	oF00_P21-C_5438_Xe	195.9	134.5	0.1

In Table S5 we report ^{129}Xe NMR chemical shifts (δ), anisotropies (CSA, $\Delta\delta$) (in ppm) and tensor symmetries η for predicted m-fluorophenol clathrate structures modelled at the screening level of theory.

Table S5: Calculated chemical shifts and CSAs at the screening level of theory for selected m-fluorophenol clathrate structures.

ID	Name	δ	$\Delta\delta$	η
mF_A	mF180_R-3_8_Xe	159.7	157.9	0.0
mF_B	mF180_R-3_1_Xe	303.3	170.8	0.0
mF_C	mF0_R-3_11_Xe	134.4	113.1	0.0
mF_D	mF0_R-3_1_Xe	240.0	125.8	0.0
mF_E	mF0_R-3_7_Xe	118.7	117.5	0.0
mF_F	mF180_R-3_5_Xe	313.2	148.1	0.0
mF_G	mF0_R-3_5_Xe	332.6	168.2	0.0
	mF0_R-3_6_Xe	202.1	73.0	0.0
	mF0_R-3_9_Xe	146.0	87.8	0.0
	mF180_119952_Xe	109.2	49.2	0.0
	mF180_R-3_11_Xe	158.5	42.9	0.0
	mF180_R-3_22_Xe	127.0	52.6	0.0
	mF0_R-3_10_Xe	123.7	35.7	0.0
	mF180_120304_Xe	233.4	95.2	0.2
	mF0_18479_Xe	158.6	81.3	0.2
	mF0_R-3_4_Xe	57.6	−0.3	0.2
	mF180_119925_Xe	132.7	86.1	0.2
	mF180_120263_Xe	130.1	−76.9	0.1
	mF180_R-3_18_Xe	157.1	152.4	0.1
	mF00_P-1_14373_Xe	161.6	−89.4	0.7
	mF00_P-1_256_Xe	104.6	−63.6	0.3
	mF00_P21_4252_Xe	210.4	−100.0	0.6
	mF0180_P-1_4959_Xe	136.5	−98.2	0.3
	mF180180_P-1_11297_Xe	137.3	71.9	0.7
	mF180180_P-1_12542_Xe	287.7	175.3	0.4
	mF180180_P-1_20220_Xe	175.9	126.2	0.6
	mF180180_P-1_22201_Xe	255.3	122.9	0.4
	mF180180_P21_3636_Xe	200.1	72.7	0.8
	mF180180_P21-C_1194_Xe	181.1	75.3	0.7
	mF180180_P21-C_8331_Xe	164.0	86.7	0.5
	mF180180_P21-C_9677_Xe	141.4	87.6	0.8
	mF180180_P21-C_9808_Xe	214.5	77.4	0.9

Table S6: Calculated ^{129}Xe chemical shifts δ and chemical shift anisotropies $\Delta\delta$ from periodic GIPAW/PBE for five likely *o*-fluorophenol clathrate structures at their PBE-TS optimized geometries. The asymmetry parameter η is zero for all structures.

Structure	δ (ppm)	CSA, $\Delta\delta$ (ppm)
*o*F_A	229.8	−38.9
*o*F_B	273.2	−86.7
*o*F_C	236.4	−15.4
*o*F_D	285.0	−46.5
*o*F_E	402.7	2.0
Experimental	256.0	−47.5

Table S7: Calculated ^{129}Xe chemical shifts δ and chemical shift anisotropies $\Delta\delta$ from periodic GIPAW/PBE calculations for seven likely *m*-fluorophenol clathrate structures at their PBE-TS optimized geometries. The asymmetry parameter η is zero for all structures.

Structure	δ (ppm)	CSA, $\Delta\delta$ (ppm)
*m*F_A	270.6	226.7
*m*F_B	295.2	216.0
*m*F_C	229.2	224.8
*m*F_D	271.4	163.4
*m*F_E	272.9	192.4
*m*F_F	314.1	203.7
*m*F_G	337.2	242.8
Experimental	228.5	183.3

10 Powder X-ray Diffraction

10.1 Sample preparation

Samples for powder X-ray diffraction (PXRD) measurements were prepared in similar conditions as the ones used in NMR experiments. Samples were placed into glass capillary tubes with 0.8 mm outer diameter, which were checked to provide low amorphous diffraction background. The capillary tubes were then sealed from one end and transferred to glass reactor tubes (4 mm outer diameter pyrex with 0.8 mm thick walls) connected to a volume calibrated vacuum line. Samples was evacuated, after which xenon was added with the help of liquid nitrogen. The amount of Xe gas was controlled with the pressure drop in the vacuum line and chosen to fill the potential voids with 3:1 (host:guest) stoichiometry and stabilise the clathrates according to available phase diagrams. Samples were flame-sealed and equilibrated for approximately two weeks at 240 K before PXRD experiments. The capillary was kept immersed in liquid nitrogen in order to preserve the clathrate phase before mounting it in pre-cooled single crystal diffractometer.

10.2 Experiments

Diffraction data was collected on a Bruker Nonius Kappa-CCD diffractometer at 100 K using graphite monochromatic Mo Kα radiation (λ = 0.71073 Å; 55 kV, 25 mA). Single frames with static samples were recorded first in order to confirm the presence of a crystalline phase and to obtain initial information about preferred orientation and size distribution of crystallites. S=The sample was rotated during PXRD measurement (exposure times between 20 to 300 s) in order to simulate randomised powder sample. Data reduction was performed with Supergui diffractometer software (single crystal to powder routine) and thus obtained PXRD patterns further analysed and fitted with Maud.[58]

10.3 Results and conclusions

10.3.1 *m*-fluorophenol

Several samples of *m*-fluorophenol were prepared, as described above. Unfortunately, no other phase than the known high density stable form (CSD refcode QAMTUU) was observed (Fig. S8). Some outgassing was observed upon melting of powder inside the capillary under a microscope but the amount of gas was rather small suggesting that the amount of actual clathrate was negligible. No change in phase or crystal texture was observed in the PXRD pattern when the temperature was raised from 100 K to 240 K (Fig. S9).

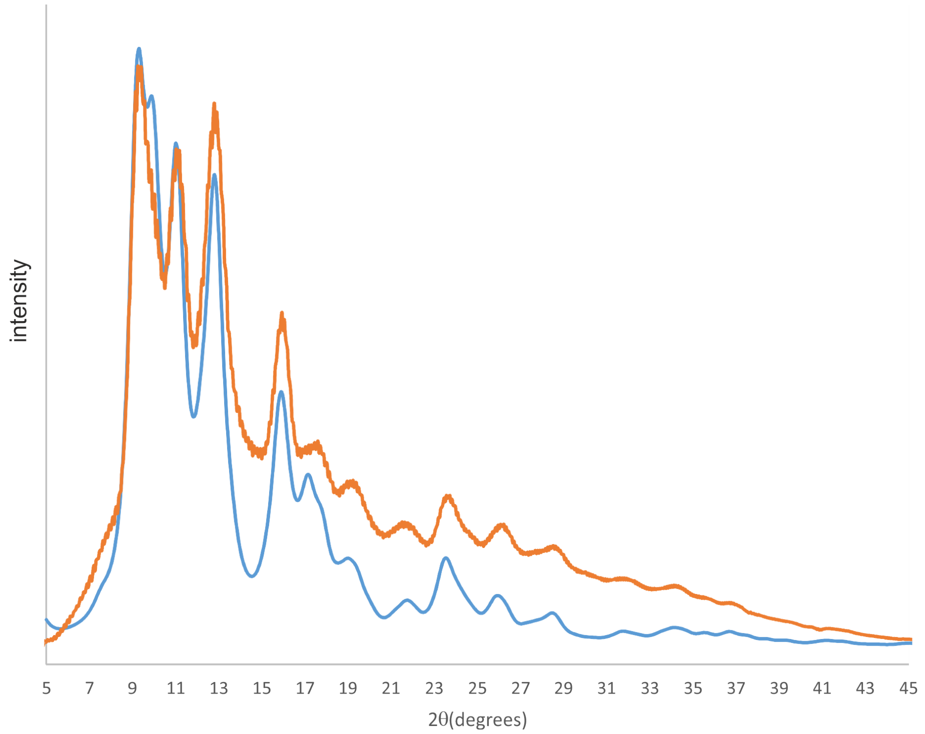

Figure S8: Experimental (orange) and simulated (blue) PXRD patterns for m-fluorophenol. The simulated pattern is for known high density stable form (CSD refcode QAMTUU).

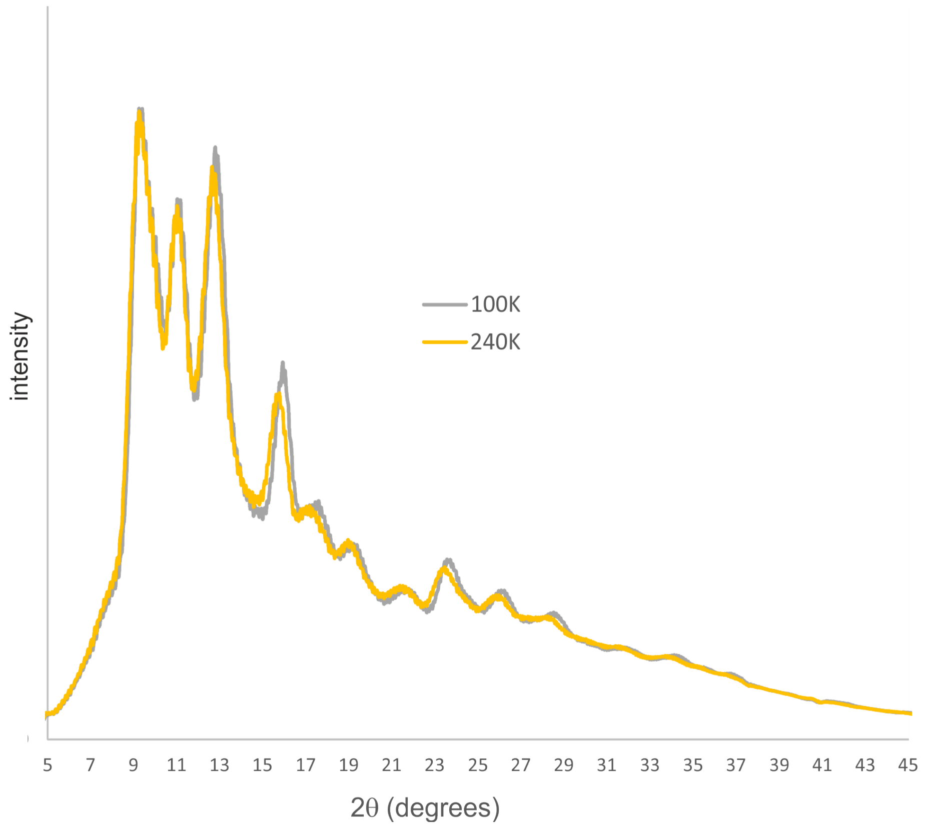

Figure S9: PXRD patterns for a sample of m-fluorophenol. Exposure at 100 K is obtained with sample freshly recovered from xenon pressurised pyrex tube and the pattern at 240 K is recorded after warming up the sample.

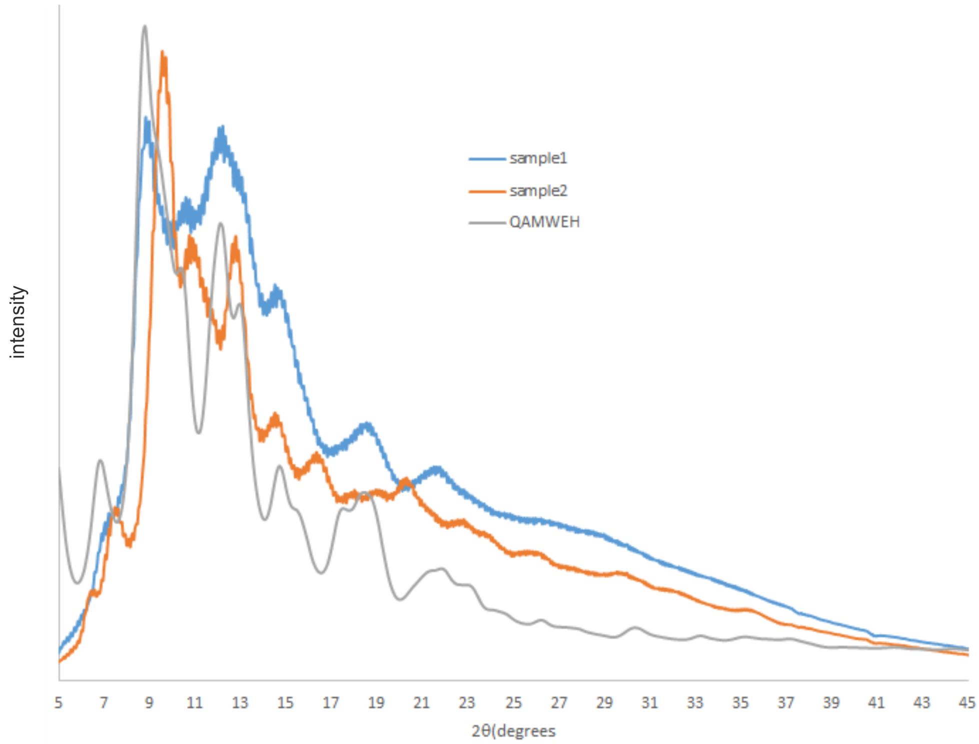

Figure S10: Comparison of simulated PXRD for the known non-clathrate phase of o-fluorophenol (CSD refcode QAMWEH, grey) with experimental PXRD from two separate samples of clathrates (blue, orange).

10.3.2 *o*-fluorophenol

The PXRD pattern of *o*-fluorophenol demonstrates the presence of a phase other than the known high density form (CSD refcode QAMWEH). Interestingly, we observe (Fig. S10) that the PXRD patterns are different when the two samples have different history (slightly different pressures, handling during the recovery of capillaries, etc.). One of these patterns (sample 1, Fig. S10) agrees with the known high density form (CSD refcode QAMWEH). The pattern of the second sample (sample 2, Fig. S10) resembles the pattern of the predicted *o*F_D structure (S11). However, the quality is not sufficient to obtain conclusive confirmation of the structure.

The presence of clathrate was confirmed by strong outgassing upon raising the temperature after the PXRD measurement. In some cases, vigorous decomposition of *o*-fluorophenol samples could be observed immediately when the temperature was raised during handling the capillaries (mounting in goniometer).

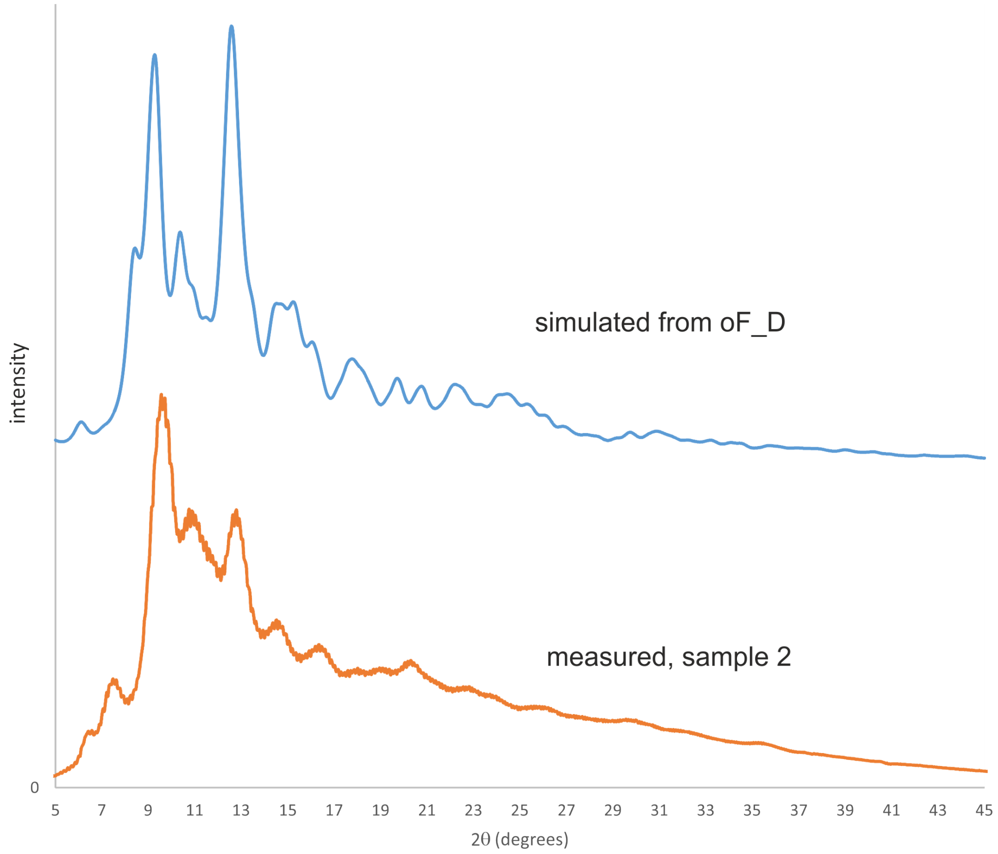

Figure S11: Comparison of the PXRD measured from sample 2 (orange) to the simulated pattern from the predicted structure (blue), *o*F_D.

Enlarged versions of Figures from main text.

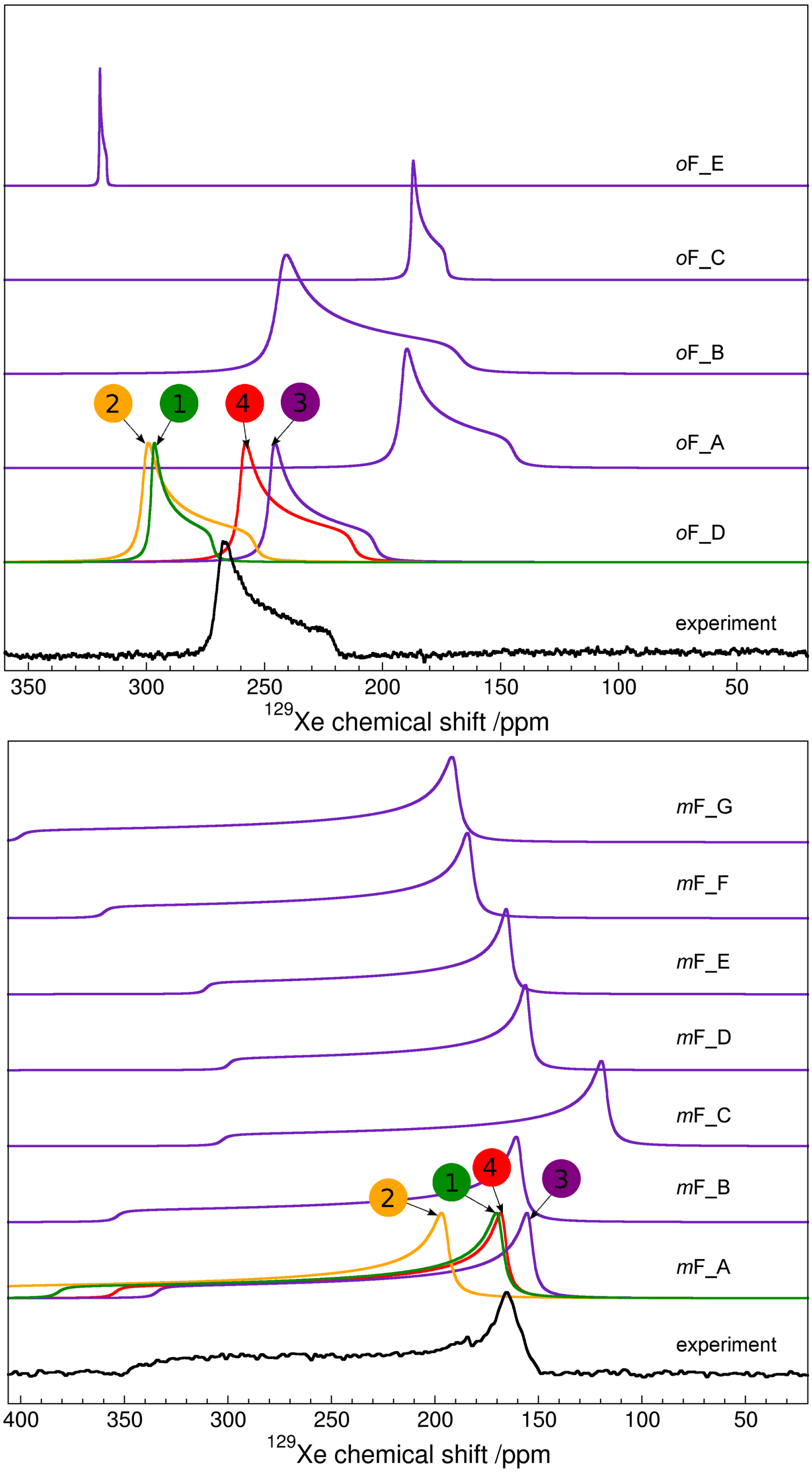

Figure S12: Experimental (black) and calculated (indigo) ^{129}Xe NMR powder spectra of o-fluorophenol (top) and m-fluorophenol (bottom) with ^{129}Xe gas reference at zero. Calculated spectra for all structures were obtained from BHandHLYP-scaled periodic PBE NMR parameters at PBE-TS optimized geometry (method/structure = BHandHLYP/PBE-TS). For the most probable candidates, oF_D and mF_A, results from different periodic modeling levels are displayed: (1, green) PBE/CSP, (2, orange) PBE/PBE-TS, (3, indigo) BHandHLYP/PBE-TS and (4, red) BHandHLYP/PBE-TS with effects due to Xe dynamics at $T = 300\,\mathrm{K}$ (see text for details).

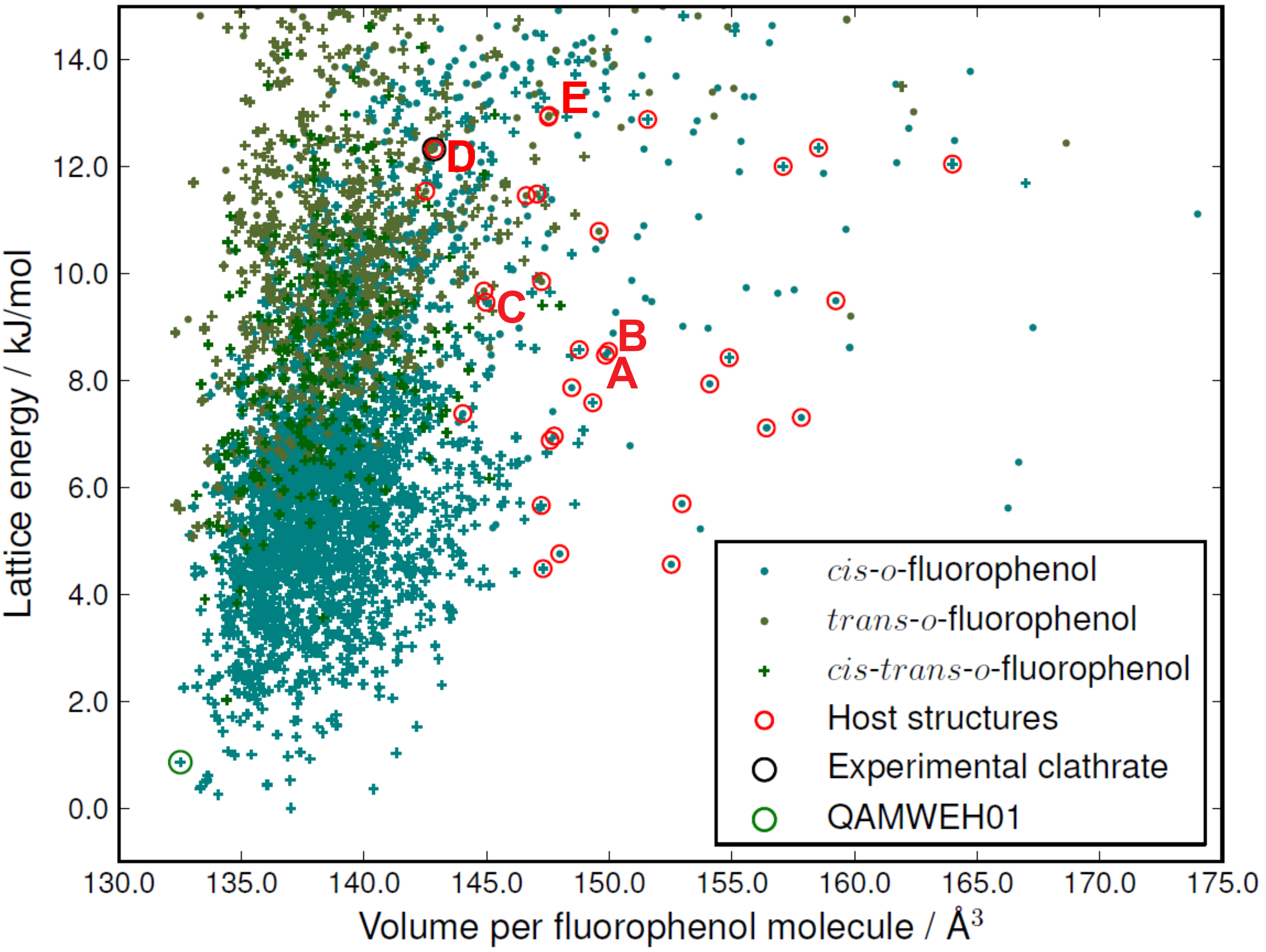

Figure S13: The crystal energy landscape of *o*-fluorophenol. Each point represents one crystal structure and is coloured according to the molecular conformation (*cis*-*trans*-*o*-fluorophenol refers to Z'=2 structures containing both conformers). Structures with a lattice energy within 13 kJ/mol from the lowest energy structure and having cavities of suitable size for xenon absorption (see text) are encircled in red. The experimentally known high-pressure polymorph (CSD refcode QAMWEH01) is encircled in green. The labels A–E correspond to structures *o*F_A to *o*F_E in the text.

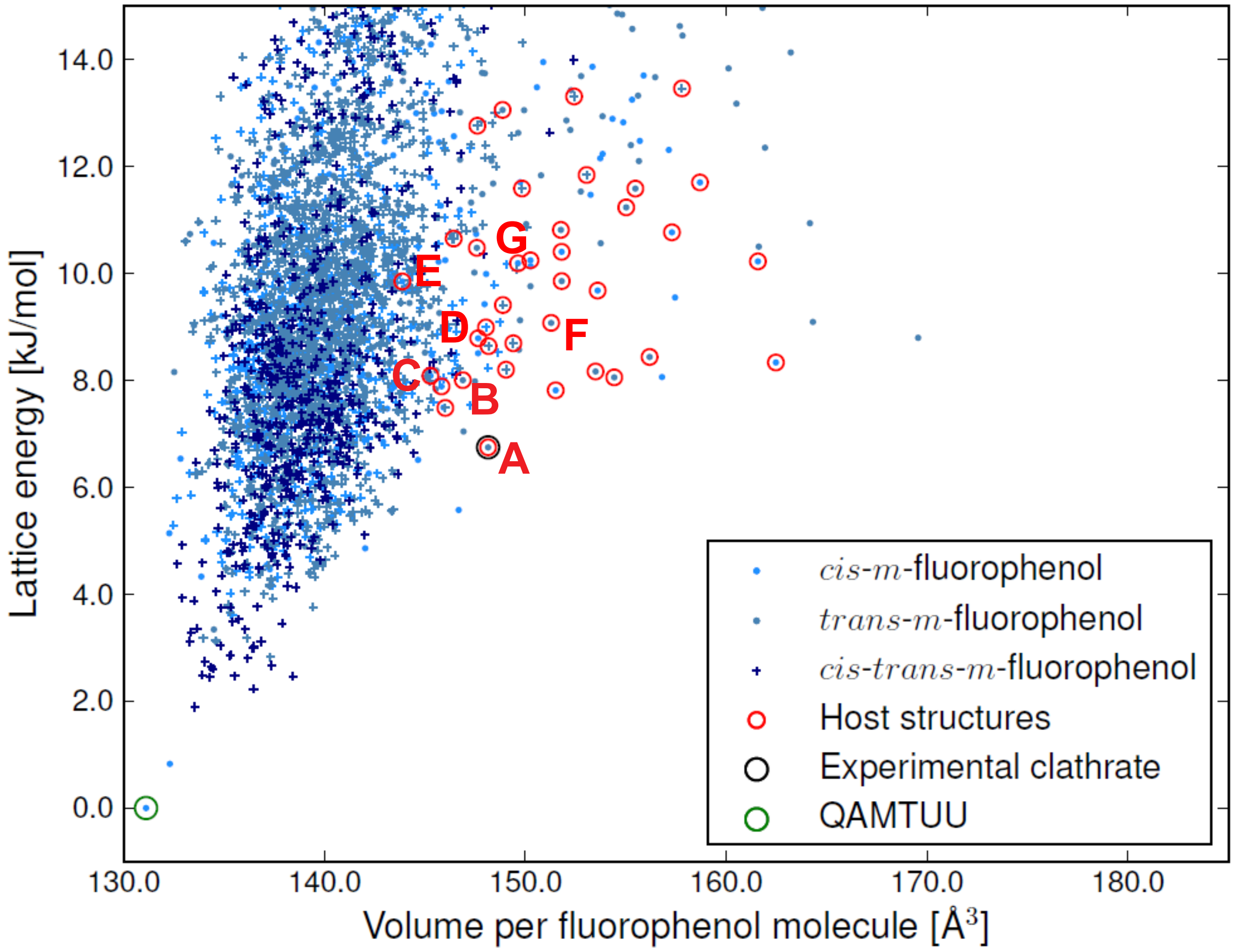

Figure S14: The crystal energy landscape of *m*-fluorophenol. Each point represents one crystal structure and is coloured according to the molecular conformation (*cis*-*trans*-*m*-fluorophenol refers to Z'=2 structures containing both conformers). Structures with a lattice energy within 13 kJ/mol from the lowest energy structure and having cavities of suitable size for xenon absorption (see text) are encircled in red. The experimentally known stable polymorph (CSD refcode QAMTUU) is encircled in green. The labels A–G correspond to structures *m*F_A to *m*F_G in the text.

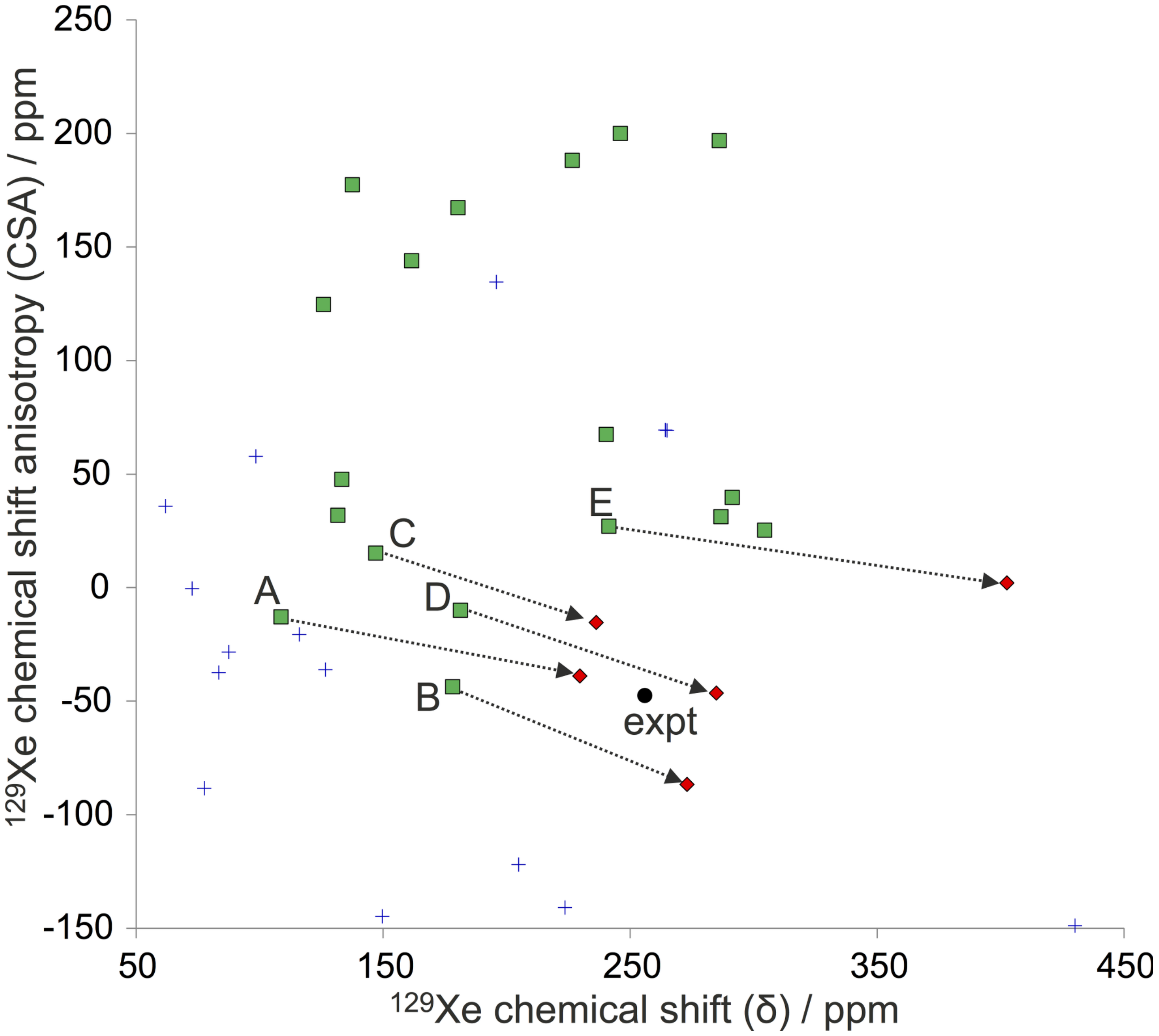

Figure S15: ^{129}Xe NMR parameters computed at screening (nonrelativistic DFT/BHandHLYP/MHA) level for cluster models of the predicted clathrate structures of *o*-fluorophenol. Structures with asymmetry $\eta > 0.04$ are shown as blue crosses, while structures with symmetric chemical shift tensors ($\eta \leq 0.04$) are shown as green squares. The experimental data (expt, black filled circle) is included for comparison. Structures that were considered for further study are connected with the black arrows to the periodic GIPAW/PBE calculation of Xe shielding from periodic PBE-TS optimized structures of the corresponding crystal (red diamonds). The labels A–E correspond to structures *o*F_A to *o*F_E in the text.

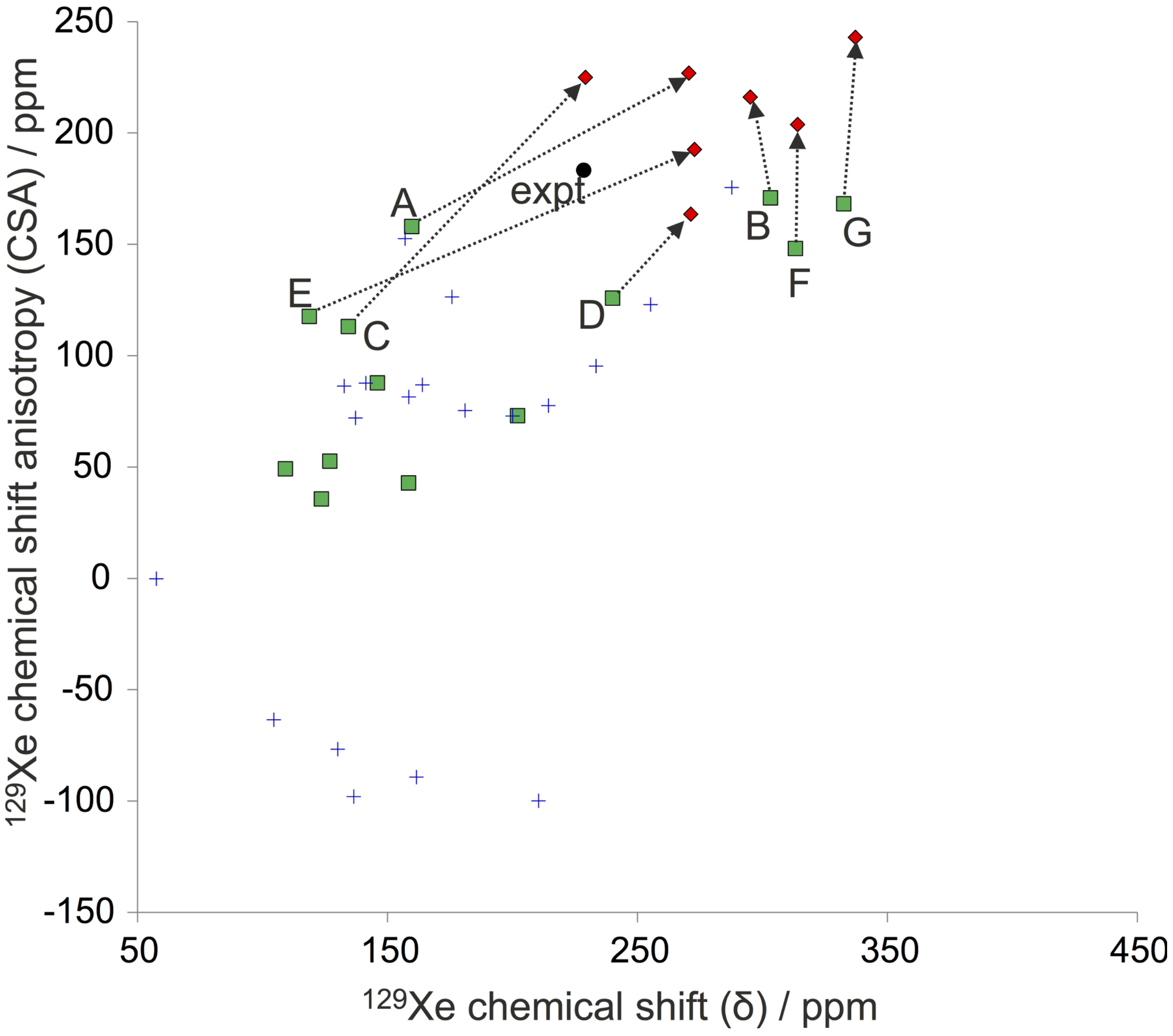

Figure S16: ^{129}Xe NMR parameters computed at screening (nonrelativistic DFT/BHandHLYP/MHA) level for cluster models of the predicted clathrate structures of *m*-fluorophenol. Structures with asymmetry $\eta > 0.04$ are shown as blue crosses, while structures with symmetric chemical shift tensors ($\eta \leq 0.04$) are shown as green squares. The experimental data (expt, black filled circle) is included for comparison. Structures that were considered for further study are connected with the black arrows to the periodic GIPAW/PBE calculation of Xe shielding from periodic PBE-TS optimized structures of the corresponding crystal (diamonds). The labels A–G correspond to structures *m*F_A to *m*F_G in the text.

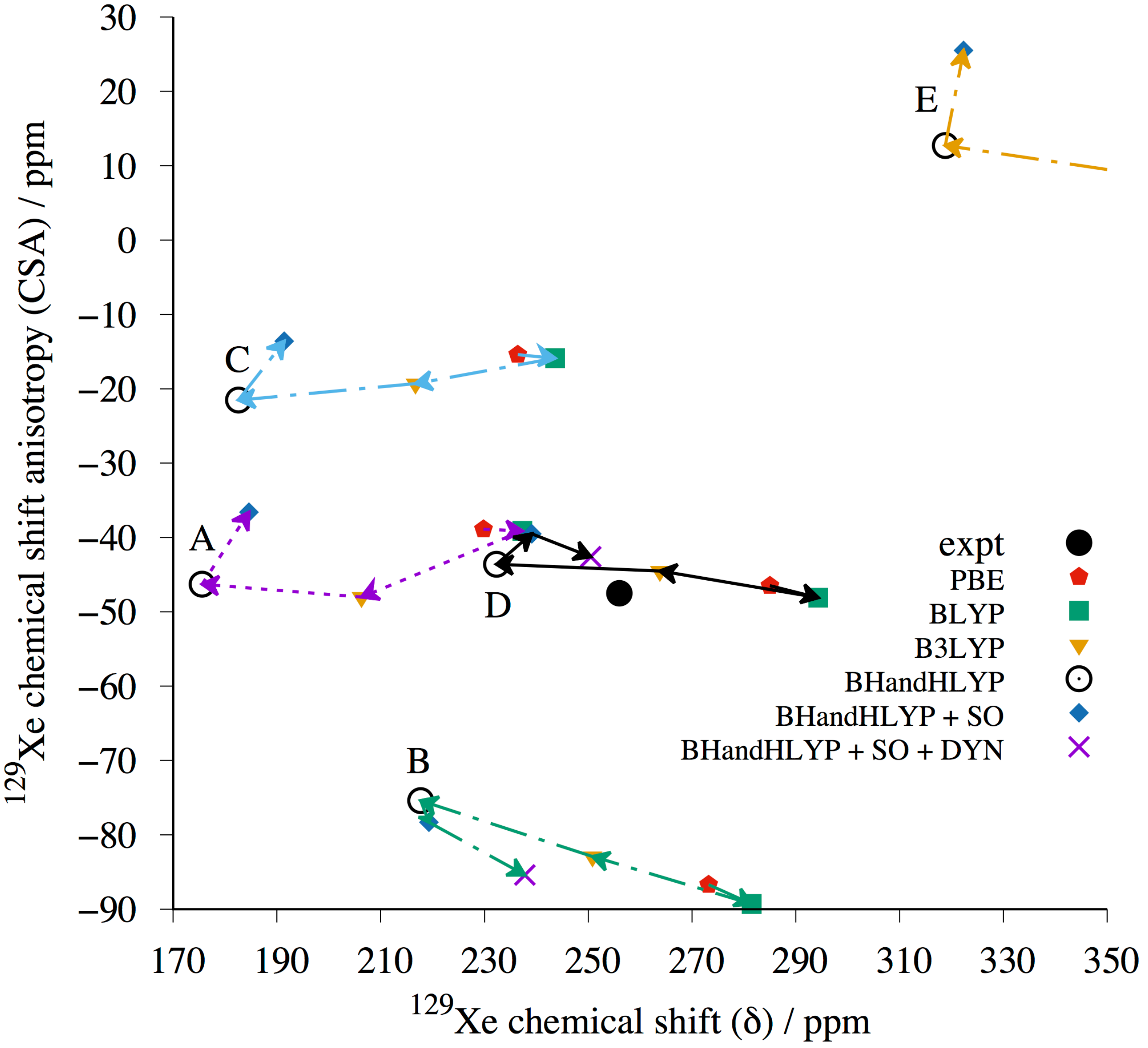

Figure S17: The periodic GIPAW results for the five most likely *o*-fluorophenol structures optimized at the periodic PBE-TS level of theory. The labels A–E correspond to structures *o*F_A to *o*F_E in the text. We expect the correct structure to approach the experimental (expt) ^{129}Xe NMR parameters, when the computed GIPAW/PBE result is scaled with factors obtained using different pure and hybrid DFT functionals with increasing amount of exact exchange in the series of PBE→BLYP(0%)→B3LYP(20%)→BHandHLYP(50%). The SO correction is added to BHandHLYP values of all structures (blue diamonds). For structures *o*F_B and *o*F_D, the final points (indigo crosses) include also the effect of Xe dynamics (DYN) at $T = 300$ K.

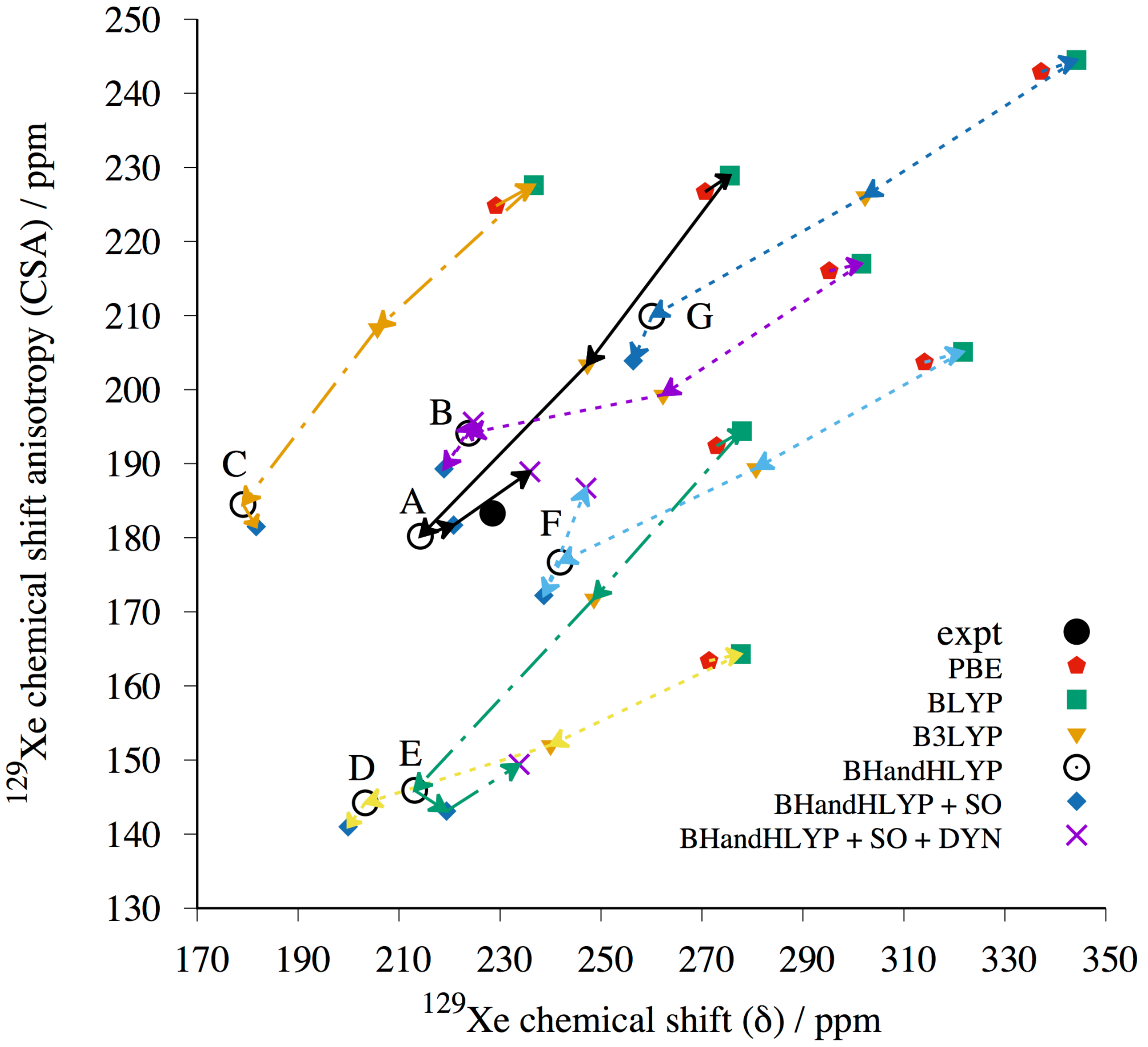

Figure S18: The periodic GIPAW results for the seven m-fluorophenol structures optimized at the periodic PBE-TS level of theory. The labels A–G correspond to structures mF_A to mF_G in the text. We expect the correct structure to approach the experimental (expt) ^{129}Xe NMR parameters, when the computed GIPAW/PBE result is scaled with factors obtained using different pure and hybrid DFT functionals with increasing amount of exact exchange in the series of PBE→BLYP(0%)→B3LYP(20%)→BHandHLYP(50%). The SO correction is added to BHandHLYP values of all structures (blue diamonds). For structures mF_A, mF_B, mF_E, and mF_F, the final point (indigo crosses) include also the effect of Xe dynamics (DYN) at $T = 300$ K.

References

[1] H. Karfunkel and R. Gdanitz, *J. Comput. Chem.*, 1992, **13**, 1171–1183.

[2] A. Kitaigorodsky, *Molecular Crystals and Molecules*, Elsevier, 2012, vol. 29.

[3] A. J. Cruz Cabeza, E. Pidcock, G. M. Day, W. D. S. Motherwell and W. Jones, *CrystEngComm*, 2007, **9**, 556–560.

[4] H. Sun, *J. Phys. Chem. B*, 1998, **102**, 7338–7364.

[5] C. M. Breneman and K. B. Wiberg, *J. Comput. Chem.*, 1990, **11**, 361–373.

[6] A. Stone and M. Alderton, *Mol. Phys.*, 2002, **100**, 221–233.

[7] A. J. Stone, *Distributed Multipole Analysis of Gaussian wavefunctions*, GMDA version 2.2.02.

[8] D. E. Williams, *J. Comput. Chem.*, 2001, **22**, 1154–1166.

[9] E. O. Pyzer-Knapp, H. P. Thompson and G. M. Day, *Acta Crystallogr. B*, 2016, **72**, 477–487.

[10] S. L. Price, M. Leslie, G. W. A. Welch, M. Habgood, L. S. Price, P. G. Karamertzanis and G. M. Day, *Phys. Chem. Chem. Phys.*, 2010, **12**, 8478–8490.

[11] G. M. Day and S. L. Price, *J. Am. Chem. Soc.*, 2003, **125**, 16434–16443.

[12] J. A. Chisholm and S. Motherwell, *J. Appl. Crystallogr.*, 2005, **38**, 228–231.

[13] A. V. Kazantsev, P. G. Karamertzanis, C. S. Adjiman and C. C. Pantelides, *J. Chem. Theory Comput.*, 2011, **7**, 1998–2016.

[14] 2014, Molinspiration property engine v2013.09, Molinspiration Cheminformatics 2014. http://www.molinspiration.com/cgi-bin/properties.

[15] H. Küppers, F. Liebau and A. L. Spek, *J. Appl. Crystallogr.*, 2006, **39**, 338–346.

[16] L. J. Barbour, *Chem. Commun.*, 2006, 1163–1168.

[17] A. L. Spek, *J. Appl. Crystallogr.*, 2003, **36**, 7–13.

[18] S. Mecozzi and J. Rebek, Jr., *Chem. Eur. J.*, 1998, **4**, 1016–1022.

[19] S. C. Nyburg and C. H. Faerman, *Acta Crystallogr. B*, 1985, **41**, 274–279.

[20] G. M. Day, T. G. Cooper, A. J. Cruz-Cabeza, K. E. Hejczyk, H. L. Ammon, S. X. Boerrigter, J. S. Tan, R. G. Della Valle, E. Venuti, J. Jose *et al.*, *Acta Crystallogr. B*, 2009, **65**, 107–125.

[21] P. Slavíček, R. Kalus, P. Paška, I. Odvárková, P. Hobza and A. Malijevskỳ, *J. Chem. Phys.*, 2003, **119**, 2102–2119.

[22] Q. Wen and W. Jäger, *J. Phys. Chem. A*, 2006, **110**, 7560–7567.

[23] W. A. Alexander and D. Troya, *J. Phys. Chem. A*, 2006, **110**, 10834–10843.

[24] Q. Wen and W. Jäger, *J. Chem. Phys.*, 2005, **122**, 214310.

[25] V. Aquilanti, D. Ascenzi, D. Cappelletti, M. de Castro and F. Pirani, *J. Chem. Phys.*, 1998, **109**, 3898–3910.

[26] P. Hobza, O. Bludskỳ, H. Selzle and E. Schlag, *J. Chem. Phys.*, 1992, **97**, 335–340.

[27] A. D. Becke, *Phys. Rev. A*, 1988, **38**, 3098.

[28] A. D. Becke, *J. Chem. Phys.*, 1993, **98**, 1378.

[29] *TURBOMOLE V6.6 2014, a development of University of Karlsruhe and Forschungszentrum Karlsruhe GmbH, 1989-2007, TURBOMOLE GmbH, since 2007; available from* http://www.turbomole.com.

[30] M. Hanni, P. Lantto, M. Ilias, H. J. A. Jensen and J. Vaara, *J. Chem. Phys.*, 2007, **127**, 164313.

[31] M. Straka, P. Lantto and J. Vaara, *J. Phys. Chem. A*, 2008, **112**, 2658–2668.

[32] S. Standara, P. Kulhánek, R. Marek and M. Straka, *J. Comput. Chem.*, 2013, **34**, 1890–1898.

[33] F. Weigend and R. Ahlrichs, *Phys. Chem. Chem. Phys.*, 2005, **7**, 3297–3305.

[34] J. P. Perdew, *Phys. Rev. B*, 1986, **33**, 8822.

[35] J. P. Perdew, K. Burke and M. Ernzerhof, *Phys. Rev. Lett.*, 1996, **77**, 3865.

[36] A. Tkatchenko and M. Scheffler, *Phys. Rev. Lett.*, 2009, **102**, 073005.

[37] S. J. Clark, M. D. Segall, C. J. Pickard, P. J. Hasnip, M. J. Probert, K. Refson and M. Payne, *Z. Kristall.*, 2005, **220**, 567–570.

[38] E. R. McNellis, J. Meyer and K. Reuter, *Phys. Rev. B*, 2009, **80**, 205414.

[39] D. Vanderbilt, *Phys. Rev. B*, 1990, **41**, 7892–7895.

[40] H. J. Monkhorst and J. D. Pack, *Phys. Rev. B*, 1976, **13**, 5188.

[41] C. Pickard and F. Mauri, *Phys. Rev. B*, 2001, **63**, 245101.

[42] J. Yates, C. Pickard and F. Mauri, *Phys. Rev. B*, 2007, **76**, 024401.

[43] N. Troullier and J. L. Martins, *Phys. Rev. B*, 1991, **43**, 1993–2006.

[44] E. van Lenthe, E. J. Baerends and J. G. Snijders, *J. Chem. Phys.*, 1993, **99**, 4597.

[45] S. K. Wolff, T. Ziegler, E. van Lenthe and E. J. Baerends, *J. Chem. Phys.*, 1999, **110**, 7689–7698.

[46] G. t. Velde, F. M. Bickelhaupt, E. J. Baerends, C. Fonseca Guerra, S. J. A. van Gisbergen, J. G. Sniggers and T. Ziegler, *J. Comput. Chem.*, 2001, **22**, 931.

[47] ADF2016, SCM, Theoretical Chemistry, Vrije Universiteit, Amsterdam, The Netherlands, http://www.scm.com.

[48] E. van Lenthe, E. J. Baerends and J. G. Snijders, *J. Chem. Phys.*, 1994, **101**, 9783.

[49] G. Schreckenbach and T. Ziegler, *J. Phys. Chem.*, 1995, **99**, 606.

[50] M. Krykunov, T. Ziegler and E. van Lenthe, *Int. J. Quantum Chem.*, 2009, **109**, 1676.

[51] J. Autschbach, *ChemPhysChem*, 2009, **10**, 2274.

[52] E. van Lenthe and E. J. Baerends, *J. Comput. Chem.*, 2003, **24**, 1142.

[53] C. Lee, W. Yang and R. Parr, *Phys. Rev. B*, 1988, **37**, 785.

[54] A. Becke, *J. Chem. Phys.*, 1993, **98**, 5648.

[55] P. J. Stephens, F. J. Devlin, C. F. Chabalowski and M. J. Frisch, *J. Phys. Chem.*, 1994, **98**, 11623.

[56] J. Roukala, J. Zhu, C. Giri, K. Rissanen, P. Lantto and V.-V. Telkki, *J. Am. Chem. Soc.*, 2015, **137**, 2464–2467.

[57] S. Grimme, J. Antony, S. Ehrlich and S. Krieg, *J. Chem. Phys.*, 2010, **132**, 154104.

[58] J. Lutterotti, *Nucl. Instrum. Methods. Phys. Res. B*, 2010, **268**, 334.
